# LiCl-induced sickness modulates rat gustatory cortical responses

**DOI:** 10.1371/journal.pbio.3001537

**Published:** 2022-07-25

**Authors:** Bradly T. Stone, Jian-You Lin, Abuzar Mahmood, Alden J. Sanford, Donald B. Katz

**Affiliations:** 1 Graduate Program in Neuroscience, Brandeis University, Waltham, Massachusetts, United States of America; 2 Department of Psychology, Neuroscience Program, and Volen National Center for Complex Systems, Brandeis University, Waltham, Massachusetts, United States of America; National Institute on Drug Abuse Intramural Research Program, UNITED STATES

## Abstract

Gustatory cortex (GC), a structure deeply involved in the making of consumption decisions, presumably performs this function by integrating information about taste, experiences, and internal states related to the animal’s health, such as illness. Here, we investigated this assertion, examining whether illness is represented in GC activity, and how this representation impacts taste responses and behavior. We recorded GC single-neuron activity and local field potentials (LFPs) from healthy rats and rats made ill (via LiCl injection). We show (consistent with the extant literature) that the onset of illness-related behaviors arises contemporaneously with alterations in 7 to 12 Hz LFP power at approximately 12 min following injection. This process was accompanied by reductions in single-neuron taste response magnitudes and discriminability, and with enhancements in palatability-relatedness—a result reflecting the collapse of responses toward a simple “good-bad” code visible in the entire sample, but focused on a specific subset of GC neurons. Overall, our data show that a state (illness) that profoundly reduces consumption changes basic properties of the sensory cortical response to tastes, in a manner that can easily explain illness’ impact on consumption.

## Abstract 2

Sickness is an internal state that impacts consumption, and so could be expected to influence the neural processing of tastes. This study shows that onset of illness changes basic properties of gustatory cortical network processing and taste responses, such that activity comes more purely to reflect the "goodness" or "badness" of tastes.

## Introduction

Neural responses in primary sensory cortex are often thought to faithfully represent physical stimuli, and while recent studies (including ours) have challenged this view by documenting enhancements and decrements in stimulus-induced firing related to animals’ internal states, there has been little work setting these changes in any sort of functional, mechanistic context. Here, we show that a state (illness) that profoundly reduces consumption changes basic properties of the sensory cortical response to tastes, and then go beyond this to precisely characterize this response plasticity, connecting it to the specific perceptual changes that drive illness’ impact on consumption.

A host of external and internal contextual variables work with experience to shape an animal’s behavior in response to sensory stimulation. Prominent among these variables are the animal’s own internal states, which can be extremely positive (e.g., “euphoria”) or negative (e.g., “depression”), and which profoundly influence the animal’s interactions with its environment [1]; systemic illness, for example, such as that induced by the intake of toxins, drastically alters an animal’s behavior in relation to food stimuli [2–4]. Such states are likely instantiated in broadly distributed neural networks, and as such, they can be indexed using spectral properties of the electroencephalogram [5,6] or local field potentials (LFPs; [7,8]).

One mechanism whereby illness might influence feeding behavior is via modifications of taste perception. Sickness changes taste palatability [9,10], making even normally preferred substances aversive (potentially prolonging infirmity by demotivating the ingestion of nutrients and/or a possible cure for said illness [11]). This impact of illness on taste palatability can be long-lasting, and even permanent [12], an intimacy of interaction that makes it reasonable to propose that illness may manifest, at least in part, as changes in the function of brain regions within which one can record palatability-related taste responses.

An obvious candidate region is gustatory cortex (GC; a fairly broad swath of granular and dysgranular insular cortex anterior to visceral cortex), which is involved both in processing of palatability and illness-related learning [13,14]. As of now, however, little is known about the cortical processing of malaise. Much is known about the basic mechanisms of function for malaise-causing agents such as lithium chloride (LiCl) at the nonneural and peripheral levels [2–4,15–19], but the impact of these basic mechanisms on activity in regions critical for chemosensory learning remains uninvestigated.

The work presented here begins to fill that knowledge gap, taking a cue from studies showing that basic information about brain states can be assessed in analyses of LFPs, and more specifically from studies showing: (1) that spectral properties of LFPs shift with changes in an animal’s internal state [20,21]; and (2) that these changes are coupled with changes in single-neuron firing dynamics [22–24]. These effects apply to GC taste coding [25], which reliably fluctuate with changes in attentional state in a manner that is specifically linked to palatability coding [26]. Here, using extracellular single-neuron recordings, we test the degree to which the onset of an illness state (indexed in terms of changes in mobility) is related to changes in GC LFPs, and go on to test how these behavioral and LFP phenomena correlate with changes in identity-related and hedonic information in GC single-neuron ensemble responses.

Our results suggest that LiCl-induced changes in GC μ (7 to 12 Hz) power reflect the onset (but not the maintenance) of sickness, in that they specifically emerge around the time at which sickness-related behaviors also emerge [2–4,27,28]. This change is accompanied by reductions in the magnitude of taste-driven responses, which convey less information about taste identity in sick rats, and simultaneously by enhancement in the palatability-relatedness of the same responses, which appear to collapse toward a simple “good/bad” judgment. These results provide evidence that emesis caused by LiCl modulates network activity in GC and plays an important role in shaping the cortical taste responses that are necessary for learning and decision-making, and further expand our insight into the state dependence of sensory coding.

## Materials and methods

### Ethics statement

All experimental protocols were conducted according to the National Institutes of Health (NIH) guidelines for animal research and approved by the Institutional Animal Care and Use Committee (IACUC) at Brandeis University (IACUC protocol # 22007).

### Subjects

Female Long–Evans rats (*n* = 26, 250 to 300 g at time of surgery; Charles River Laboratory, Raleigh, North Carolina) that were naïve to tastants served as subjects in this study. Animals were maintained on a 12-h light/dark schedule, with experiments performed in the light portion of the cycle from 7:00 to 11:00 AM. Rats had ad libitum access to food and water except during experimentation (see below).

### Apparatus

Neural recordings were made in a custom Faraday cage (21 × 24 × 33 cm) connected to a PC and Raspberry Pi computer (Model 3B). The Pi controlled the opening time and duration of solenoid taste delivery valves. The PC controlled and saved electrophysiological recordings taken from electrode bundles via connections to an Intan data acquisition system (RHD2000 Evaluation System and Amplifier Boards; Intan Technologies, LLC, Los Angeles). Each bundle consisted of 32 microwires (0.0381 mm formvar-coated nichrome wire; AM Systems) glued to a custom-made interface board (San Francisco Circuits) and soldered to a 32-channel Omnetics connector, which was fixed to a customized drive [29].

### Surgery

Rats for recording experiments were anesthetized using an intraperitoneal (IP) injection of a ketamine/xylazine mix (1 mL ketamine, 0.05 mL xylazine/kg body weight). Maintenance of anesthesia followed 1/3 induction dose every 1.25 h. The anesthetized rat was placed in a stereotaxic frame (David Kopf Instruments; Tujunga, California), its scalp excised, and holes bored in its skull for the insertion of self-tapping ground screws and an electrode bundle. Each bundle was painted with a lipophilic membrane stain (Thermo Fisher Scientific, Indianapolis, Indiana) and inserted 0.5 mm above GC (coordinates: AP +1.4 mm, ML ±5.0 mm, DV −4.4 mm from dura)—a location corresponding primarily to layer V of the granular and dysgranular segments of the insular midsection, in which we have reliably found taste responses in the past (see [30–32]). Assemblies were cemented to the skull, along with 2 intraoral cannulae (IOCs; flexible plastic tubing inserted close to the tongue in the cheek and extending upward to the top of the skull) using dental acrylic [26]. The rat’s body temperature was monitored and maintained at approximately 37°C by a heating pad throughout the duration of the surgery. Rats were given 6 d to recover from the surgery, after which electrode bundles were lowered by 30 to 50 μm/d until discriminable GC neuron waveforms were visible in multiple channels; there they stayed through the entire protocol, which lasted (at most) a single session of each type (saline and LiCl; see below).

### Water restriction

**Fig 1:**
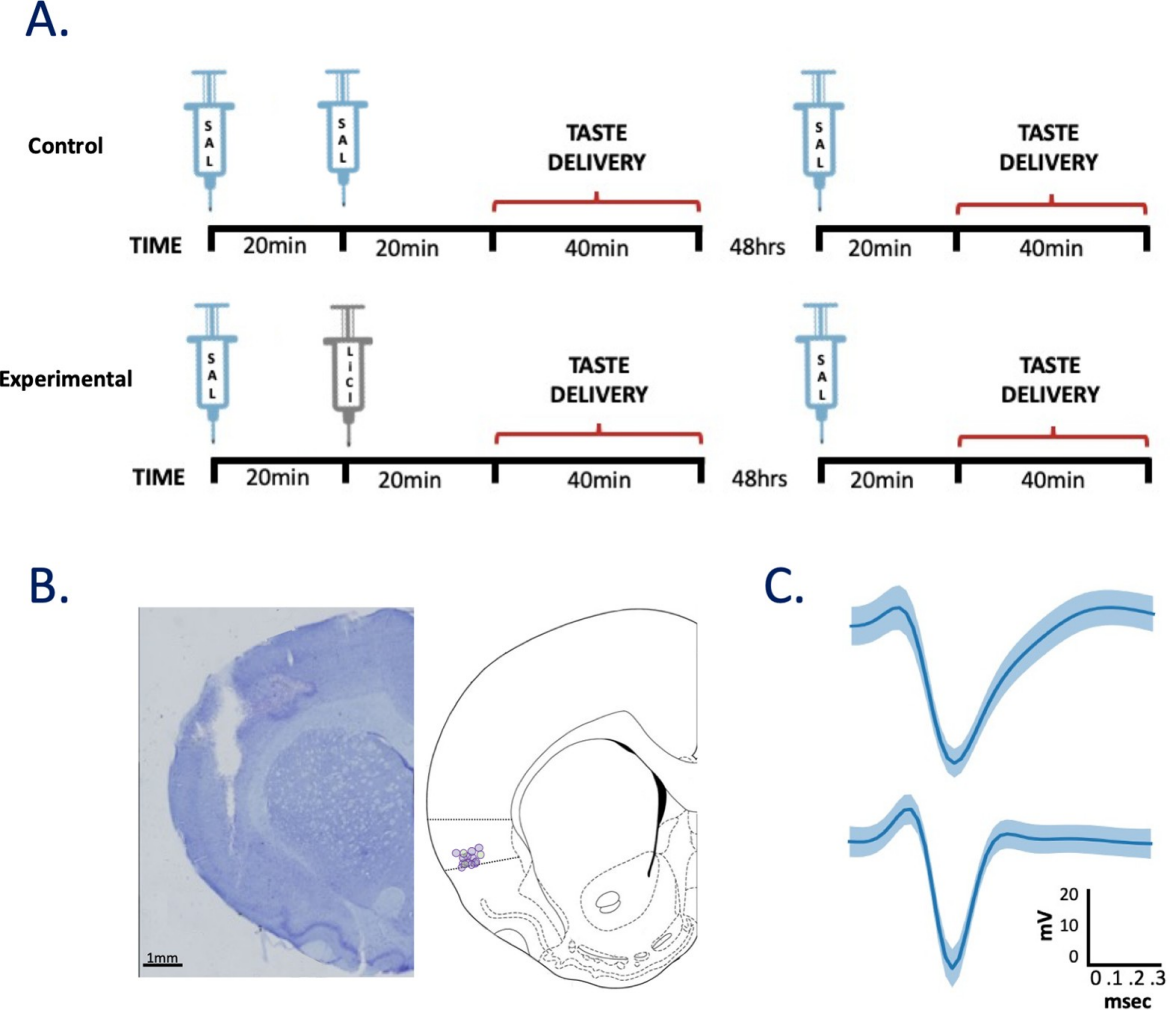
General sickness induction protocol and histological verification of recording location in GC. ** (A)** On Day 1, rats received a single injection of saline followed by either an injection of saline (Control Group; top) or 0.15 M LiCl (Experimental Group, bottom). GC activity was recorded for 20 min following each injection, after which tastes were delivered via IOC across an approximately 40 min session, and 48 h later, only a single saline injection was administered to both groups, 20 min before the taste testing period. **(B)** A coronal slice (left) from 1 rat, stained with cresyl violet. The track is an electrode bundle lesion, with the end of the lesion marking the final resting location of the wires. A coronal slice schematic (right) with circles indicating the electrode bundle resting location for each rat (experiment 1: purple, experiment 2: green). **(C)** The average waveform of a representative putative pyramidal cell [top] and interneuron [bottom]. Light blue shading represents the standard deviation of the noise in the waveform. GC, gustatory cortex; IOC, intraoral cannulae; LiCl, lithium chloride.

During experimentation (Fig 1A), mild water restriction (ad libitum access to 20 mL every day at 4 PM, 6 h after experimental sessions), started 6 d following surgery, ensured engagement in the task. Daily records were kept of weight and water intake to ensure that rats did not fall below 85% of presurgery weight.

### Experimental design

On the second day of water restriction, rats began 2 d of habituation to liquid delivered directly to the tongue via IOC, with 60 and 120 30-μL infusions of water delivered each day, respectively. To maximize our ability to record from neurons held stably across both (saline and LiCl) sessions, electrodes were lowered to their recording depth (approximately 4.5 mm DV from dura) on the second day of habituation and left at that depth.

For Experiment 1, on the morning following the second habituation session, in vivo recording sessions commenced: Rats received a subcutaneous (sc; 0.50% of body weight) injection of isotonic saline followed 20 min later by an injection of either a second saline injection (Control) or illness-inducing LiCl (Experimental; **Fig 1A**); following each injection, neural and behavioral data were collected—20 min of “passive” recording followed by 120 taste delivery trials in which 1 of 4 gustatory stimuli (0.1 M sodium chloride [NaCl], 0.3 M sucrose, 0.1 M citric acid [CA], and 0.01 M quinine-HCl [QHCl]) was delivered via IOC in a pseudo-random order. Stimuli and concentrations were chosen to ensure a range of distinct taste identities and palatabilities and to maximize comparability to our [33–36] and others’ [37,38] studies. A second testing session, identical to the first session with the exception that only a single sc. injection of saline was administered 20 min prior to taste delivery, was given 48 h after the first testing session (**Fig 1A**), with the electrode bundle remaining in the same place. To test whether any observed LiCl/saline differences were not order effects, a subset of animals (*N* = 5) were subjected to the same experimental protocol with the exception of the order of injections being reversed (2 saline injections on the first day, LiCl injection on the second). Our analyses revealed no difference between the orders *(ps* > 0.05).

For Experiment 2, additional (*N* = 3) taste-naive animals were subjected to a single recording session in which the injection of LiCl was immediately followed by taste deliveries via the IOC.

### Illness induction

Because we wished to elicit a mild illness that would not cause large scale, overt behaviors that might serve as confounding explanations for any observed changes in neural activity, we induced malaise using a mild concentration of LiCl (0.15 M, delivered sc). Multiple studies demonstrate that the malaise induced using this concentration is strong enough to cause conditioned taste aversion (CTAs) without causing gross changes in behavior [4,39].

### Behavioral analyses

The assessment of lethargy provided a basic measure of sickness [2,28]. Three cameras (positioned below, above, and diagonally within the behavioral chamber (see Apparatus)) captured mobility in a separate subset of animals implanted only with IOCs (*N* = 4 and 5, Control and Experimental). Each video was manually scored for lateral (grid-line crossings) and vertical (rears; 2 forelimb paws off the ground for >0.5 s) movements by a trained, but blind to condition, observer. Such events have been shown to be sensitive measure of welfare in rodents—healthy, nonanxious mice/rats will perform lateral and vertical movements as a means to explore their environment [19,40]. Given the small size of the recording chamber, the rats had limited ability to move laterally, which led us to focus our analyses primarily on vertical movements. Healthy/sick differences in frequency and duration of these movements were analyzed and fit with sigmoid functions so that we could determine whether and when LiCl-treated rats became ill.

### Histology

At the completion of the experiment, rats were deeply anesthetized with ketamine/xylazine (200:20 mg/kg, IP) and then perfused transcardially with physiological saline followed by 10% formalin. The brains were extracted and stored in a 10% formalin/30% sucrose solution for at least 3 d before staining, after which they were frozen and sliced on a sliding microtome (Leica SM2010R, Leica Microsystems; thickness 60 μm). Slices were mounted, and sections were stained with a Nissl stain (Thermo Fisher Scientific, Indianapolis, IN) to evaluate cannulae and electrode tracks, respectively, via inspection of fluorescence on a light microscope (**Fig 1B**).

### Electrophysiology methods and analyses

Differential recordings from the micro-electrodes were sampled at 30 kHz using a 32-channel analog-to-digital converter chip (RHD2132) from Intan Technologies. The signals were digitalized online at the head stage/amplifier and saved to the hard drive of the PC. The collected recordings were filtered for either single-neuron action potential isolation (300 to 3,000 Hz bandpass) or LFPs (1 to 300 Hz lowpass) and analyzed off-line. Putative single-neuron waveforms (3:1 signal-to-noise ratio) were sorted using a semi-supervised methodology: Putative action potentials were grouped into clusters by a Gaussian mixture model (GMM) with strict noise tolerance and refined manually; conservatism was enhanced via a requirement that the variability in spiking be random (and thus not reflecting biased cluster cutting; for the details of the recording system and Python scripts, see [29]). To further ensure that the isolated waveforms represented single-neuron records, we also calculated the interspike intervals and removed units for which ≥1% of the intervals disobeyed the biological constraints of refractory period. By this approach, a total of 266 and 190 GC neurons were isolated from, respectively, 7 Experimental (saline-LiCl) and 5 Control (saline-saline) rats across 24 sessions (2 sessions/animal).

All analyses, unless otherwise stated, were performed using all neurons; in a few cases (noted in the text), analysis was restricted to neurons that were taste responsive (see also Table 1).

**Table 1:** Animal assignment in each figure.

Figure	Animals	Neurons	Experiment: Detail
Fig 2 Panel A Panels B/C	1 Sal-LiCl 5 Sal-Sal vs. 7 Sal-LiCl rats	N/A	LFP
Fig 3 Panel B	4 Sal-Sal vs. 5 Sal-LiCl rats	N/A	Behavioral verification
Fig 4 Panel C	7 Sal-LiCl rats	Sal (*n* = 136) LiCl (*n* = 174)	Single unit spiking activity
Fig 5	7 Sal-LiCl rats	Sal (*n* = 136) LiCl (*n* = 174)	Single unit spiking activity
Fig 6 Panels A/B	7 Sal-LiCl rats	Sal (*n* = 136) LiCl (*n* = 174)	Single unit spiking activity
Fig 7 Panel B	3 LiCl rats	56 taste-responsive units	Single unit spiking activity
Fig 8 All panels	7 Sal-LiCl rats	44 held units	Single unit spiking activity

**Fig 2:**
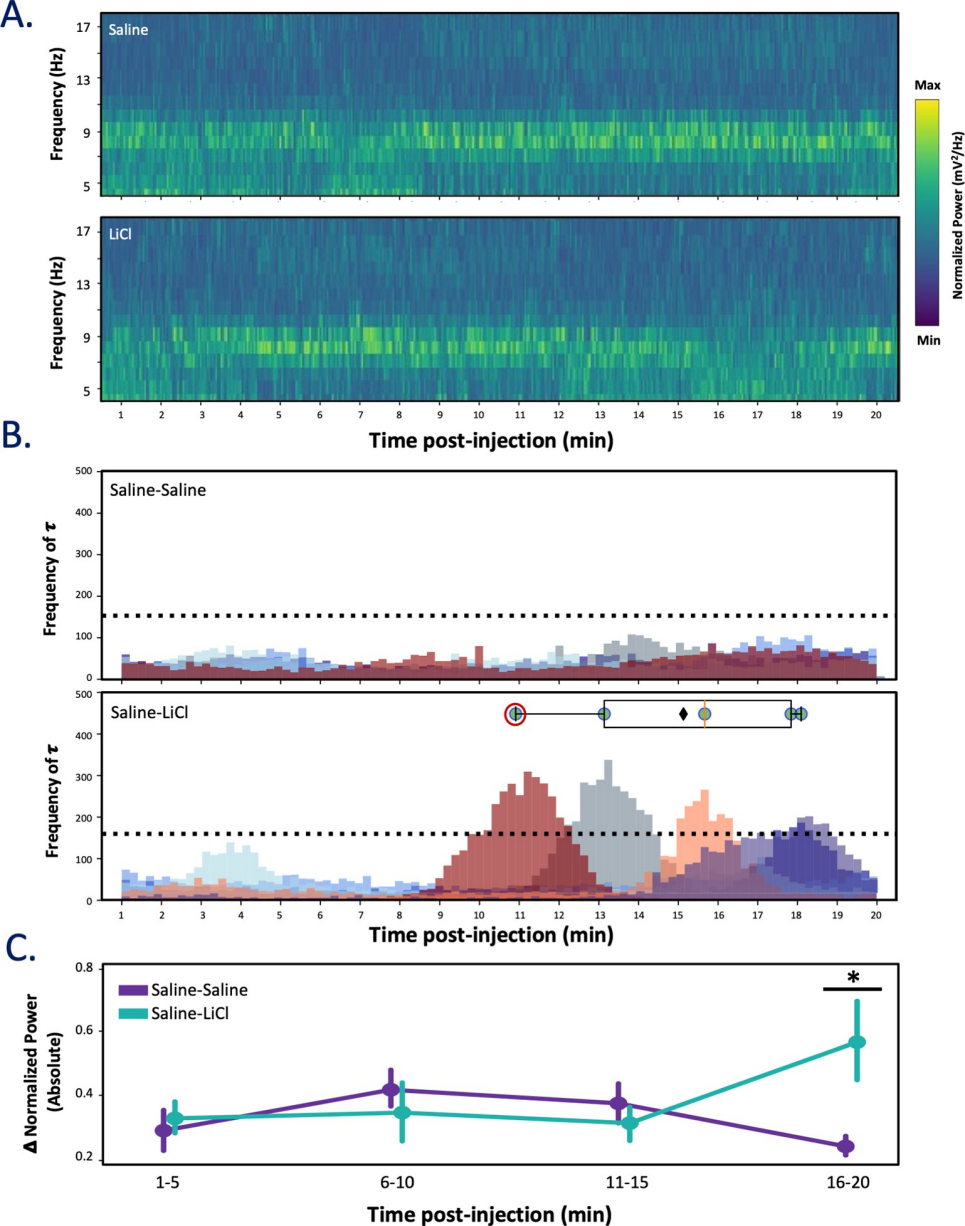
LiCl administration alters GC LFP 15-min post-injection. **(A)** Representative spectrograms from 1 animal (saline-LiCl condition) showing activity after saline (top) or LiCl (bottom) injection. LiCl reduces power in μ during the last half of the 20-min period. **(B)** The posterior distributions for a single change point model identify the probabilities of a change (shown as frequency of τ) in μ power (each color represents distribution for single animal) for saline-saline (top, *N* = 5) and saline-LiCl (bottom, *N* = 7) groups. The horizontal dashed lines indicate the 99th percentile from temporally shuffled data, and the overlaid boxplot depicts the range in peak likelihood of change in μ power for distributions with values higher than the 99th percentile: Each dot represents a single animal (the animal from panel A is noted with a red circle); the box extends from the lower to upper quartiles (red line: median, black diamond: mean) of likelihood. The mean onset of the change in μ power occurs at 15.11 min, with all change points occurring after 10-min post-injection and before 18-min post-injection. **(C)** The absolute difference in μ for saline-saline (purple) and saline-LiCl (teal) groups reveals a significant interaction between change in μ power and quartile (*p <* 0.05, 2-way ANOVA), with LiCl changing μ power significantly within the fourth quartile (* *p* < 0.05; 1-way ANOVA). The error bars represent SEMs. Data underlying this figure can be found in S1 Data. LFP, local field potential; LiCl, lithium chloride.

The same experimental data (all neurons recorded from the experimental group (Fig 1A: Experimental [Saline-LiCl; Sal-LiCl]; main dataset) were used for Figs 2 and 4–6 with additional control data (Fig 1A: Control [Saline-Saline; Sal-Sal]) were also used in Fig 2. Fig 7 involved collection of a new dataset from 3 rats that received LiCl injection prior recording. Fig 8 used all neurons that were held across sessions from the main dataset. LFP, local field potential; LiCl, lithium chloride.

LFPs were extracted only from channels containing isolated single-neuron data, in order to avoid potential signal artifact arising from noisy/broken channels. Data were averaged across electrodes within and across session for each animal, and analyzed across the 1 to 20 Hz frequency bands, with focus on the μ bands (7 to 12 Hz, [41–43] in 1-min time bins). After normalizing (across the entire session) each animal’s μ power to between 0 and 1, the LiCl vectors were subtracted from the saline vectors to reveal changes in μ following LiCl administration, and an ANOVA was used to compare groups. Differences were considered significant only if 3 or more consecutive time bins reached a *p* < 0.05 criterion.

### Change point analysis

To determine the position of a change point in GC μ power, we used a custom model implemented in the PyMC3 probabilistic programming package [44], with parameter estimation performed using Markov Chain Monte Carlo sampling (see details below). The LFP power time series were z-scored, and then modeled using 2 normal distributions; the model attempts to detect a single change point (tau) in the mean value of the LFP power as the time series switches between distributions. Change points were determined for each animal independently.


$$LFP\;Power\;Mean:\;\mu_{1},\mu_{2}\;\sim N(0,1)$$

LFPPowerVariance:σ∼HalfCauchy(1)

ChangepointPosition:τ∼Uniform(0,20)

$$Observed\;LFP\;Power:\;Obs\;(t)∼\left\{ \begin{array}{c} N(\mu_{1},\sigma )\;t\;<\;\tau \\ N(\mu_{2},\sigma )\;t\;\geq \;\tau \end{array}\right.$$

Since sampling returns a distribution over the parameter values explaining the data, distributions of tau with low variance (high peaks) indicate strong presence of a change point. To mark the position of these change points using the tau distribution in an unbiased manner, we compared the tau distributions (containing equal numbers of samples) of our time series (actual data) to those of 50 temporally shuffled time series (shuffled data), which, by definition, have no change points. Points in the actual data distribution higher than the 99th percentile of the shuffle distribution were marked. The average of these marked time points was taken to be the position of the change point for that time series.

The calculation of the behavioral changes and LFP change points was performed on separate cohorts of animals. We then quantified the likelihood that the close synchronization between the behavioral and neural changes seen in our data could have happened by chance, by comparing the data to simulated data produced under the assumption that the latencies of the average behavioral and LFP change points are drawn from independent, uniform distributions. We calculated the distribution of summed, absolute distances between the behavioral and LFP change points, testing the hypothesis that the change points in our data are clustered more tightly than those drawn from the simulation (a *p*-value less than 0.05 indicates a significantly more coupled relationship than if time points were truly independent).

### Taste responsiveness

We defined basic responsiveness to taste delivery by first subtracting the across-trial averaged 1 s of prestimulus firing from the first 1 s of taste-evoked firing for each neuron (ignoring the first 200 ms of post-taste activity; see below), and subjecting the resultant single-neuron data to a Mann–Whitney U test to determine whether a significant change in firing rate occurred when the taste hit the tongue. These data were then pooled to ascertain the overall magnitude of response change under different conditions. But because taste delivery could reduce firing to below baseline levels in some neurons (thereby masking the true magnitude of effects in the averaging of enhanced and reduced firing rates), we divided the sample into those in which firing normalized to pre-stimulus baseline was greater than zero, and those in which it was less than zero. We refer to these as “excitatory” and “inhibitory responses,” respectively, but note that this designation only refers to the direction of firing rate change—we make no claims regarding whether or not “inhibitory responses” are caused by direct inhibitory influence on these neurons; nor do we intend to imply that these are responses found in inhibitory interneurons. We used a 2-way ANOVA to test whether LiCl administration changed taste response magnitude.

Note that in these analyses, as well as in those described below, we typically ignored the first 200 ms of post-taste activity. This was done because we have repeatedly observed that these early responses are seldom chemosensory in our paradigm, reflecting only tactile stimulation of the tongue (see [26,30–32,36,45]). This differentiates our paradigm from those in which rats perform active licking or lever-pressing for fluid delivery [46–50], wherein researchers observe a much shorter pre-chemosensory response, likely because the rodent is able to anticipate taste acquisition (see also [51]).

### Taste specificity

The above analysis is performed on data averaged across tastes, and therefore serves only to estimate whether, and how, GC is responsive to oral stimuli—not whether that response depends upon stimulus identity. To determine how LiCl impacted the taste specificity of GC responses, we subjected whole ensembles to a standard linear discriminant analysis (LDA) classifier used in previous studies [30,32,52]. This classifier tests the reliability with which a trial of taste-evoked responses in an ensemble of simultaneously recorded neurons can be identified among responses to other tastes. We binned neural responses into 250 ms bins and used a linear classifier with a leave-one-out validation approach, calculating the prediction accuracy of the classifier averaged across each excluded trial for each time bin. Paired-sample *t* tests (with Bonferroni correction) were performed for each post-taste delivery epoch (see above) to determine the impact of LiCl.

To assess changes of taste discriminability within a single session, the ensemble-wise LDA classifier was trained on the first 5 trials (per taste) and tested on later trials. Here, and when testing the degree to which sickness impacted discriminability in real-time, we restricted the analysis to taste-responsive units (typically >70% of the total number of recorded neurons [30,31,45]; see **Table 1**): Across-iteration averages were computed for each tested trial and the resulting means were binned into 5-trial blocks (approximately 6 min/block). Blocks were normalized (between 0 and 1) across animals, and results were subject to a repeated measures ANOVA and a series of paired-sample *t* tests (with Bonferroni correction).

Finally, it is worth noting that taste responsiveness and taste specificity provide slightly different types of information about GC activity. It is possible for a neuron to be taste responsive and not respond distinctly to different tastes, and it is possible for a neuron to respond in a manner that is taste specific without the average, across-stimuli response being significantly modulated from baseline. Both are useful.

### Taste palatability

Using our now-standard palatability-correlation analysis [32,35,51,53], we evaluated the degree to which LiCl altered the amplitude of palatability-relatedness in late epoch GC taste responses. Using a moving window (window size: 250 ms, step: 25 ms), we correlated firing rates with well-established palatability ranks (sucrose > NaCl > CA > QHCl; [32,54]) and compared the magnitude of this correlation in healthy and sick rats.

In a further analysis of palatability-relatedness, we computed what we termed a “pure palatability index” (PPI), adapting methods from [55] to test the degree to which GC taste responses could be described to code a simple “good versus bad” dichotomy. Specifically, we compared the Euclidian distances between single neurons’ responses (normalized to −500 ms pre-taste delivery) to tastes with similar palatability (i.e., sucrose and NaCl, CA and QHCl) to those between tastes with different palatability (sucrose and CA, sucrose and QHCl, NaCl and CA, NaCl and QHCl). This amounts to evaluating the ratio of the distances between “different-palatability” and “same-palatability:” a lack of palatability-related information results in a PPI of 0 (because tastes of similar palatability and tastes of different palatabilities are equally distant from one another); the more polarized the response into “good versus bad”—the larger the ratio—the more positive the PPI. To avoid artificially attenuating the effect via the inclusion of information from different epochs in single averages, the PPI was calculated from firing within the middle of our standard epochs [30,31]. The results were significance tested using a Wilcoxon test.

We hasten to emphasize that these analyses of the degree to which firing is palatability-related are quite different from the above-described analysis of the degree to which firing is taste specific. In fact, taste information and palatability-relatedness are actually very different variables—one makes use of any cell-specific differences in responses to different tastes, and one is specifically measuring whether a particular pattern of responsiveness exists—necessitates these differences in analysis.

### Stability of single-neuron waveforms across days

A subset of analyses required the stable tracking of neurons held across testing sessions (i.e., “held neurons,” **Table 1**). To evaluate whether a neuron was “held,” we performed a spike shape analysis the likes of which has been brought to bear in several previous studies [33,35,56,57]. We applied a conservative criterion for stability based on within-session data (comparing each neuron’s waveforms from the first third of the session to those of the last third)—only neurons for which the between-session (nonparametric clustering statistic) value was less than the 95th percentile value calculated from the within-session data were deemed to have been stably held. Of the entire population of recorded GC neurons, a total of 44 (23.2%) satisfied this criterion.

### Cluster detection in patterns of healthy-ill response differences

A clustering analysis was used to identify groups of held neurons for which LiCl impacted palatability-related firing similarly. A condition response was determined for each held unit (under each condition) by taking the average (minmax-normalized) pre- (−750➔250 ms) and post-delivery (250➔750 ms) firing rates (presented as a percentage of maximum responsiveness). We then calculated and plotted the distance and Cartesian direction between condition responses or each neurons’ taste response, yielding 176 (44 held units × 4 tastants) response differences (RDs)—quantification of how LiCl administration changed the excitatory or inhibitory response to the taste. As a conservative estimate of RD likeness, we determined the number of clusters that best fit a GMM probability distribution (calculating the Bayesian information criterion) as done previously [58]. Classifications were considered valid only if they fell within the 95% confidence interval from the centroid of each respective cluster (see **Fig 8D**), after which we constrained our analyses to neurons within the same cluster.

### Cluster-specific taste palatability

Using methods described above, we evaluated the effect of LiCl administration on palatability-related firing for neurons within clusters. A nonparametric 2-way ANOVA was used to reveal whether differences (saline–LiCl) in hedonic coding (Spearman *rho*^*2*^) differed across cluster and time during taste processing.

## Results

### LiCl administration changes GC LFPs and behavior

We recorded activity for 20 min following subcutaneous injections of either LiCl or saline, before beginning to record taste-driven activity (**Fig 1A**); GC single-unit responses (**Fig 1C**) and LFPs were acquired from drivable bundles of 32 wires. Given that network function, measured in terms of spectral properties of LFPs, appears to change with even the most innocuous of body states (e.g., sleep versus wake, [59,60]), and that single-neuron firing is altered in concert with these changes [61,62], our investigation into the characterization of illness started with assessment of changes in GC LFPs. We focused on power in the mu (μ: 7 to 12 Hz; [25]) range, because power in this frequency band has proven particularly sensitive to changes in even general states related to wakefulness and attention [20,25,26,41,63]. While the results described below were also observed in frequency ranges above (e.g., β) and below (i.e., θ) μ, they were centered on and largest in the μ range.

Save for the brief period immediately following handling and injections, the amplitude of μ in the LFPs remained relatively stable across the first half of the (20 min) post-injection recording periods for both experimental groups, but an impact of LiCl injections emerged in the second 10-min period following injections for the saline-LiCl group (**Fig 2A:** LFPs spectral power from a representative rat following either saline [top] or LiCl injection [bottom]). The precise nature of this impact varied with individual, in some cases (*N* = 3) involving a reduction of μ power and in some cases an increase (*N* = 4), but a change point analysis quantifying the time points and likelihoods of change in μ power for each animal (across both experimental groups) revealed that LiCl-induced changes in GC μ power reached significance at around the same time in the majority (5 out of 7) rats (**Fig 2B, bottom**). That is, the timing of the change in spectral properties was remarkably reliable across the 7 rats, despite the fact that in some cases the change involved an enhancement of the power in the μ range, and in some cases the opposite: had no change occurred, or had the change been gradual/subtle following injection (as in saline-saline–injected rats, **Fig 2B, top**, *N* = 5), the likelihood of change (τ; posterior distribution) would be spread uniformly across post-injection time; instead, the analysis reveals that a change in GC μ power reliably occurs between 10.89 and 17.07 min post-injection (never before and never after), with the mean likelihood occurring at 15.11 min.

An analysis of the group data confirms these rat-by-rat results, demonstrating that absolute changes in μ power following LiCl injection emerged late in the 20-min post-injection recording session (compared to saline-saline animals, **Fig 2C**). A 2-way ANOVA on these data revealed a significant interaction between group and quartile (*F*(3, 33) = 3.62, *p* = 0.023); a subsequent 1-way ANOVA confirmed that the absolute difference reached significance only in the 16 to 20-min post-injection bin (*F*(1, 11) = 5.14, *p* = 0.04) for the saline-LiCl group. While in some cases, an apparent change in spectral power (in this case, μ power) could in fact be an artifactual effect of firing rate changes [64], our investigation failed to observe concomitant changes in firing rate at any time across the 20-min post-injection recording session (*F*(3, 33) = 2.39, *p* = 0.09; see also **Fig 8A** below); this suggests that our observed effect on μ is not simply a reflection of firing rate change, but rather a matter of network synchrony being modulated.

Overall, these results accord well with those of previous studies, in that they demonstrate: (1) that changes in LFP activity are hallmarks of the onsets of cortical state changes, regardless of the specific directionality of the changes [65–68]; and (2) that sickness-related behaviors such as immobility emerge at approximately this time point following LiCl injections [19,40,69–72].

**Fig 3:**
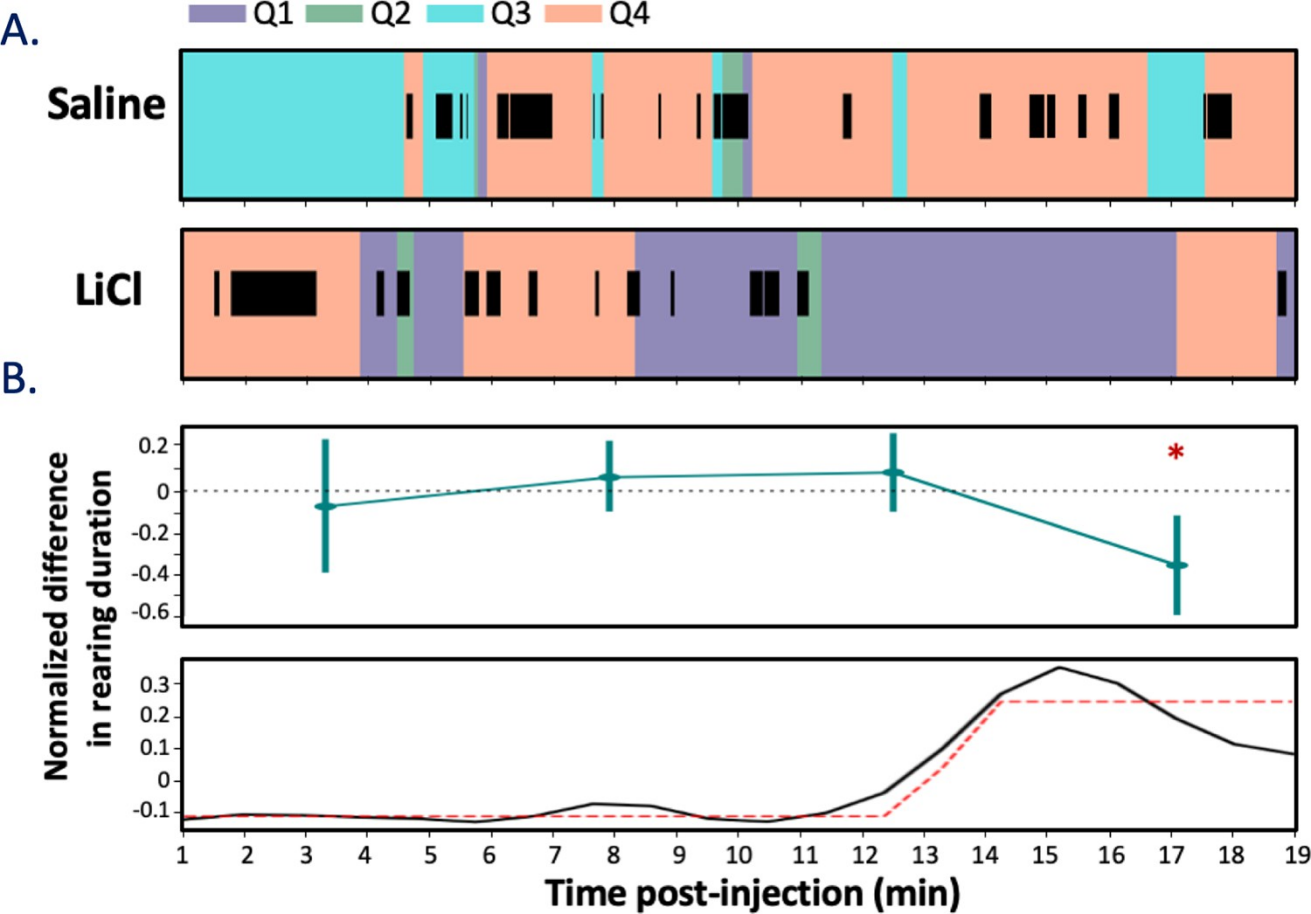
LiCl administration reduces rearing approximately 12 min after LiCl injection. **(A)** An example rat’s movement through time around the cage space (color coding shows the rat’s position in the box, Quadrant 1–4) overlain with rearing (black bars) durations after saline (top) and LiCl injections (bottom). **(B)** The average normalized LiCl–saline difference in rearing duration across animals (*N* = 4 and 5, saline and LiCl) reveals (top) a significant reduction of rearing occurring in the fourth quarter of the session (* *p* < 0.05, Wilcoxon-rank sum). A more fine-grained analysis (bottom) suggests that the change in behavior (black line) begins at 12-min post-injection; the dashed red line illustrates the sigmoidal fit *(*r^2^ *=* 0.72). The error bars represent SEMs. Data underlying this figure can be found in S2 Data. LiCl, lithium chloride.

To more completely test this last point, we compared our LFP data to (independently collected and blindly coded) video recordings of illness-related behaviors, predicting that the above-described changes in network activity would roughly coincide with times at which changes in mobility appeared following LiCl injections. Because we specifically injected a low concentration of LiCl in order to avoid gross movement changes that could confound interpretation, and because the small recording chamber limited the rats’ ability to move laterally, we focused our measurement of mobility on rearing events in which the animal lifted both forepaws off the floor simultaneously without proceeding to grooming [73,74]. We specifically hypothesized that reduction of such rearing events, which has been linked to mild LiCl-induced illness [72], would occur at around the time that we observed changes in cortical LFP μ power (**Fig 3A**).

In fact, the durations of rearing events declined following LiCl (*N* = 5) injection, but not following saline injection (*N* = 4). A 2-way repeated measures ANOVA brought to bear on the 20-min recording session (again binned into 5 min quartiles to ensure sufficient power; **Fig 3B, top**) revealed a significant difference in rearing duration emerging post-injection (*F*(3, 182) = 3.14, *p* < 0.05)—a change that, like the change in cortical LFP power, became significant (according to post hoc Wilcoxon signed-rank tests) only in the 15- to 20-min post-injection bin (W = 158, Z = 0.65, *p* = 0.024, *r* = 0.50), compared to rats receiving only saline injections.

A sigmoidal curve fit to the normalized LiCl-saline difference in rearing duration (**Fig 3B, bottom**) confirmed that the appearance of this illness-related change in behavior (the asymptote minima of fit; r^2^ *=* 0.72) aligned well with the above-noted change in GC μ power (compared to **Fig 2, top**), again suggesting that the onset of LiCl-induced illness is reflected in the function of the GC network. The fact that the **Figs 2 and 3** data were collected from separate groups of rats only increases the conservatism of this interpretation—the likelihood that this close alignment of behavioral and neural changes would occur if LFP change points of the first cohort and behavioral change points of the second cohort were random (uniformly distributed) and uncoupled is extremely low (difference between real and modeled data, *p* = 0.03). While this result does not conclusively rule out the possibility that LiCl could independently cause illness and cortical changes (see Discussion), the pattern of results implicates the emergence of illness (as reflected in behavior) in the change in GC spectral properties.

### LiCl administration causes GC taste responses to collapse into a “good-bad” distinction between tastes

**Fig 4:**
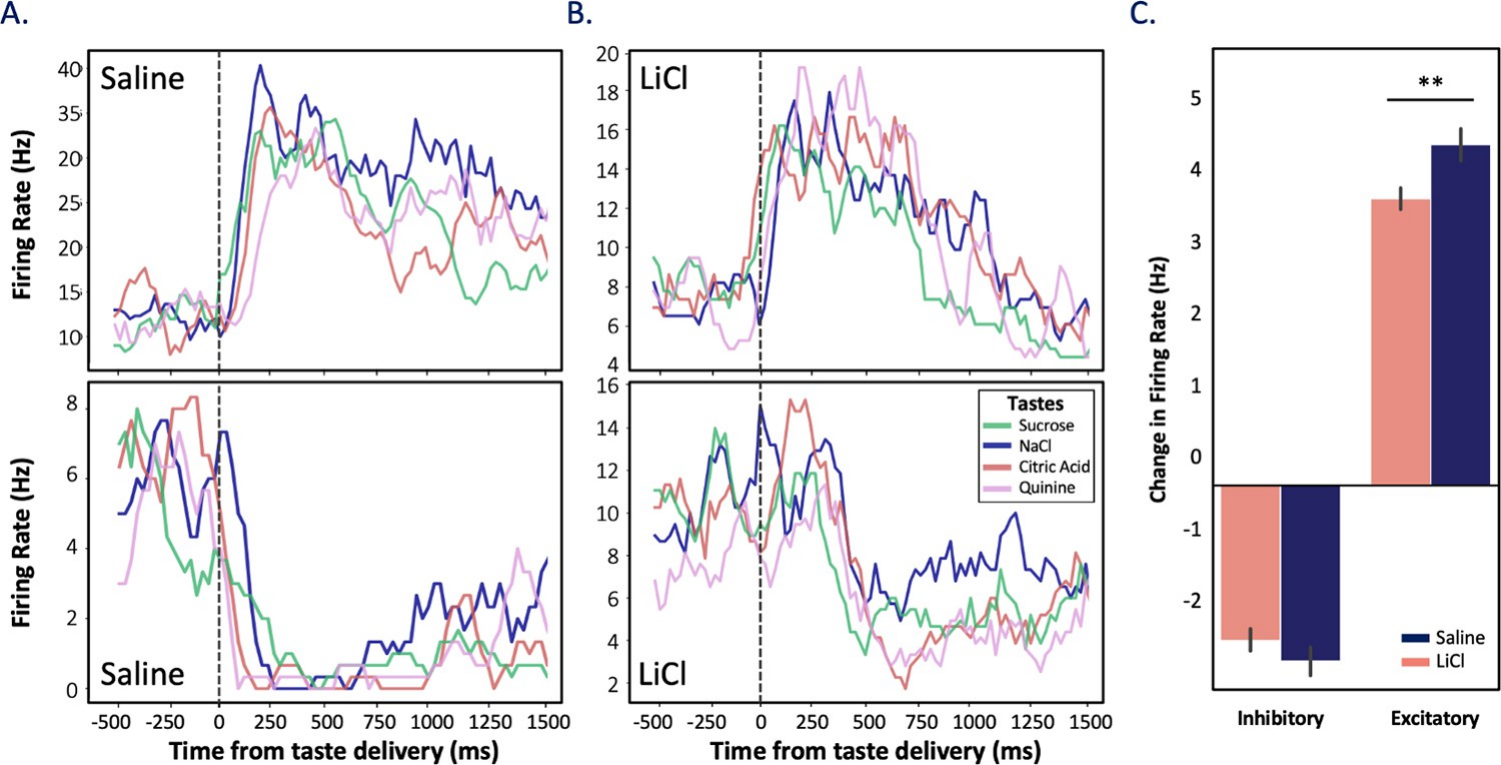
LiCl administration reduces magnitudes of taste responsiveness. PSTHs of trial-averaged firing rates of 4 representative neurons recorded in (A) saline and (B) LiCl states. The examples in the top panels show excitatory responses, and the bottom examples show inhibitory responses. Vertical dashed lines indicate the time of taste delivery. Subtle variances in firing rates observed between 0–200 ms are not statistically significant (1-way ANOVA, ps > 0.05, see Methods). (C) Illness decreased the magnitude of these responses; for neurons with excitatory responses, this reduction was significant (** *p* < 0.01; 2-way ANOVA with Tukey post hoc). Error bars represent SEMs across neurons. Data underlying this figure can be found in S3 Data. LiCl, lithium chloride; PSTH, peristimulus time histogram.

**Fig 4A** shows 2 example of GC single-neuron taste responses—one for which taste administration caused inhibitory firing rate changes, one excitatory—from each type of session (saline/healthy and LiCl/illness). As an initial look at how illness impacts these responses, we evaluated the magnitudes of responses elicited by tastes delivered directly into the mouth via IOC. While initial paired-sampled *t* tests (with Bonferroni correction for multiple comparisons) performed across the entire pooled sample failed to reveal an impact of LiCl, this apparent non-effect proved to be an artifact of averaging across neurons with “excitatory” and “inhibitory” taste responses: When we performed separate analyses of those 2 types of taste responses, it became clear that LiCl-induced illness reduced the magnitude of both excitatory and inhibitory GC taste responses (**Fig 4B**). A 2-way ANOVA performed on these data revealed a significant interaction between the condition and taste response direction (*F*(1,3191) = 6.05, *p* = 0.014); a Tukey post hoc comparison showing the main effect of illness was larger for excitatory responses (*p* = 0.003) but not for inhibitory responses (*p* > .05).

**Fig 5:**
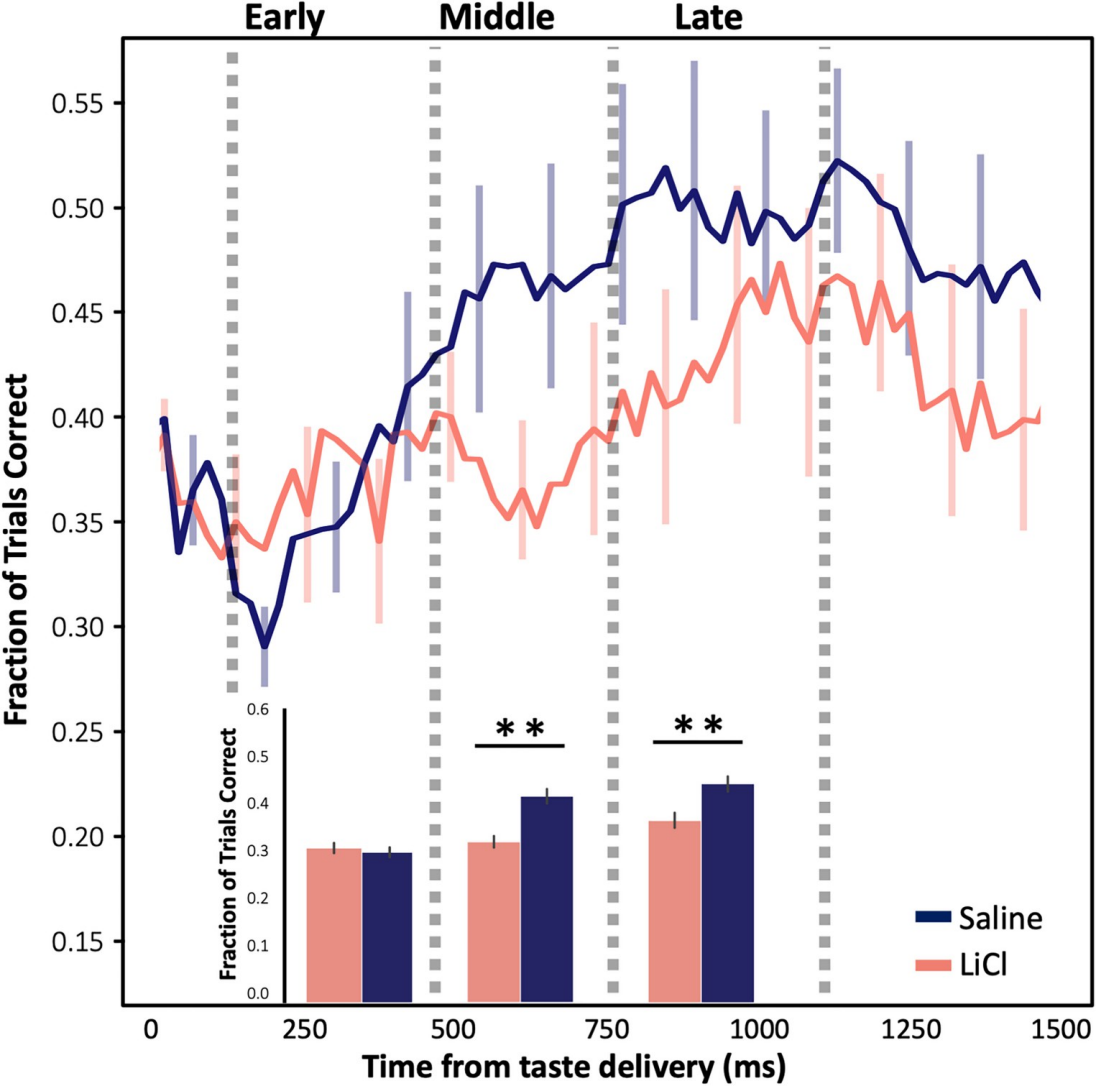
LiCl administration reduces taste discriminability in neural ensembles. The time course of trials in which the taste could be correctly identified by ensemble firing, according to LDA, shows reduced discriminability of responses when an animal is sick (LiCl; coral, *n* = 174) in comparison to when they are healthy (saline; purple, *n* = 136). The vertical dashed lines delineate approximate epochal boundaries, as defined in previous research; collapsed into these epochs (bottom bars, the effect of illness only becomes significant within the middle (400–700 ms) and late (800–1,100 ms) epochs post taste delivery (** *p <* 0.01; pairwise *t* test with Bonferroni correction). The error bars represent SEMs. Data underlying this figure can be found in S4 Data. LDA, linear discriminant analysis; LiCl, lithium chloride.

The above analysis, however, examines only responsiveness, providing no information regarding whether the impact of LiCl-induced illness differs for different specific tastes. We therefore performed an LDA, quantifying the reliability with which GC responses to one taste could be differentiated from responses to other tastes. As shown in **Fig 5** (blue trace and bars below), LDA enables us to correctly identify administered tastes from middle epoch responses on over 40% of the trials in healthy sessions (well above chance, which = 25%); this percentage quickly rises to over 50%, despite the use of a small time bin sliding window analysis that left the data at the mercy of unsmoothed trial-to-trial variability. More to the point, this distinctiveness (i.e., classifiability) of responses was significantly diminished following systemic LiCl administration (coral trace and bars in **Fig 5**), with the decrement becoming significant in the “middle epoch” and continuing into the “late epoch” (Mann–Whitney *U* (1-tailed) = 1,952, *M* = 34.06%/43.77%, SD = 11.09%/14.58%, *p* = 0.0003; *U* = 2,045, *M* = 39.78%/47.83%, SD = 15.72%/14.64%, *p* = 0.001, respectively). Taste responses carry less identity-related information when the rat is ill.

The fact that this decrement in discriminability extends into the “late” epoch led us to ask whether LiCl-induced illness might also change the palatability-relatedness of coding as well (a feature that is known to emerge at this point in GC taste responses; see [30,31]). We began with the simple hypothesis that reduced discriminability should imply reduced palatability-relatedness, testing this hypothesis in the standard manner [32,34,51,53]—i.e., calculating moving window correlations between firing rates and the known canonical palatability ranks of the administered taste stimuli.

**Fig 6:**
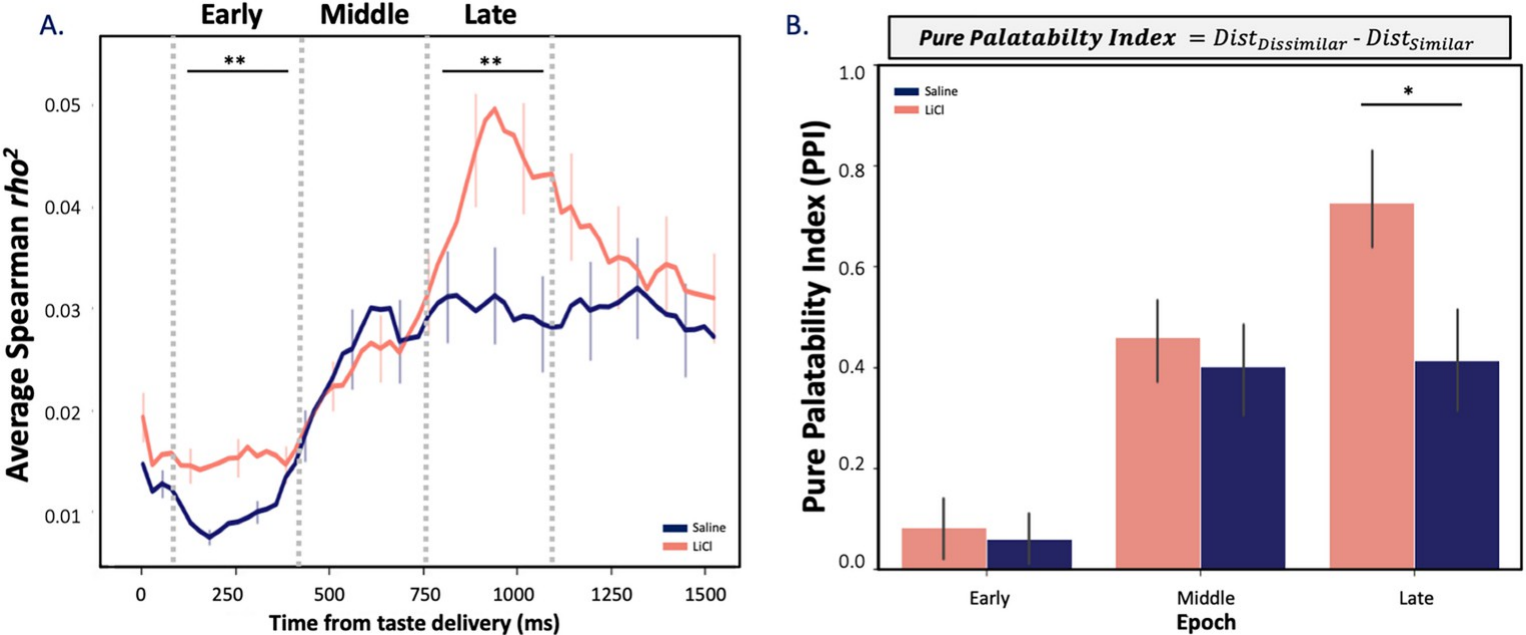
LiCl administration shifts GC taste responses in the direction of a simple “good vs. bad” judgment. **(A)** The time course of average correlations (Spearman rank-correlation coefficient) between firing rates and known palatability shows the expected increase as responses reached the late epoch, but this rise reached a higher peak following LiCl (*n* = 174) than saline injection (*n* = 136, Kruskal–Wallis H-test, ** *p <* 0.01). **(B)** The sickness-induced reduction of taste discriminability, juxtaposed with this sickness-induced enhancement of palatability-relatedness, suggests that illness selectively decreases the distinctiveness of tastes with similar palatability. As predicted, a PPI, which evaluates this possibility (see inlaid equation), peaks at a larger late epoch value following LiCl injection than following saline (unpaired 2-sample Wilcoxon test; * *p* < 0.05), reflecting sickness-induced polarization of coding. The error bars represent SEMs. Data underlying this figure can be found in S5 Data. GC, gustatory cortex; LiCl, lithium chloride; PPI, pure palatability index.

As has been observed many times previously, palatability-related information in our GC taste responses climbed (regardless of condition) across the period leading into the late epoch (**Fig 6A**; note that we currently have no explanation for the small, low magnitude, but significant saline-LiCl session difference in palatability-related firing in the earliest responses, but see Discussion); despite the well-known, oft-commented upon variability of sensory neural activity [75,76], this property of GC taste responses shines through reliably in assessments of large numbers of neurons and across multiple animals. The climb calculated following saline and LiCl injections diverged significantly; however, particularly as the late epoch was reached—peak correlations following LiCl injections were higher than those observed in saline condition within this epoch (H(1) = 8.68, *p* = 0.003). This means that our initial hypothesis was disconfirmed: Whereas illness reduces taste response discriminability, it increases late epoch taste response palatability-related content.

The above results beg the question “how can illness reduce information pertaining to identity while enhancing the palatability-relatedness of the same taste responses?” We hypothesized that these seemingly contradictory results could be reconciled if (and only if) illness causes GC coding to collapse toward a simple “good versus bad” judgment, preferentially decreasing the differences between the coding of tastes with similar palatabilities—making sucrose and NaCl (the palatable tastes) responses more similar and making quinine and citric acid (the aversive tastes) more similar—and leaving GC responses closer to a simplistic, “pure” code of whether or not a taste is palatable. We tested this hypothesis by quantifying the distance in Euclidean space between neural responses for all pairs of similar (e.g., sucrose and NaCl) and dissimilar (e.g., sucrose and quinine) taste stimuli.

Specifically, we calculated what we term a “pure palatability index”—the difference of the distances (*Dist_Dissimilar_*−*Dist_Similar_*) between responses to “different-palatability” and “same-palatability” tastes. By this analysis, the more polarized the response into “good versus bad”—the larger the difference—the more positive the PPI; a PPI of one (or greater) would indicate that GC neural responses are determined purely on the basis of whether the taste in question is pleasant or aversive. **Fig 6B** shows the results of this analysis. One-sample *t* tests comparing each PPI to a null result revealed that the PPI reaches significance only after the early epoch in both conditions (*ps* < 0.01); meanwhile, a Wilcoxon signed-rank test (performed because the data differed significantly from normal, *p* >0.05) revealed that LiCl-induced illness significantly enhances the PPI in the late epoch (W = 68379, Z = 0.54, *p* = 0.037, *r* = 0.09), reflecting enhanced polarization of the coding of palatable and aversive tastes. Thus, our second hypothesis was confirmed: Illness shapes the coding of taste hedonics by enhancing the polarization between similar and dissimilar tastes in the late epoch GC taste responses, and thereby simultaneously increases the overall palatability correlation and reduces the average discriminability of the responses.

### Illness-related changes in taste coding occur in single neurons

The above results suggest that ensembles of GC neurons assayed approximately 20 min after LiCl administration code tastes differently than ensembles of GC neurons assayed in healthy rats. The implication of these results is that coding in individual neurons changes as sickness emerges, but the analyses above provide only indirect evidence for this implication; they stop short of directly testing whether single-neuron responses truly change with the onset of illness.

**Fig 7:**
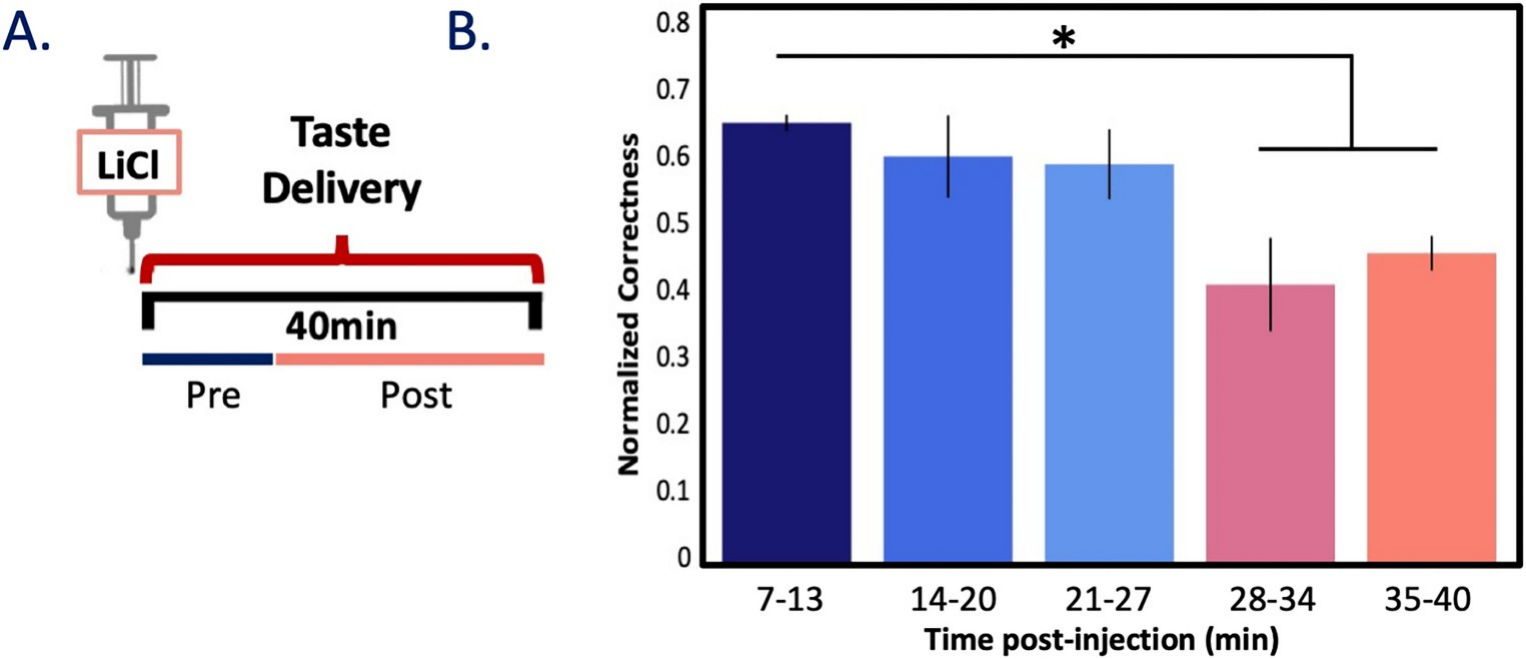
Reduction in taste discriminability emerges with sickness within session. **(A)** A schematic of the second testing protocol, in which an injection of 0.15 M LiCl was immediately followed by delivery of tastants via IOC for approximately 40 min. **(B)** Bar plot shows the percent of trials in which the proffered taste was correctly identified using an LDA (trained on the first 5 trials) from GC responses. Across *N* = 3 animals (*n* = 56 taste responsive neurons), taste discrimination is significantly reduced post-sickness induction. * *p <* 0.05; paired *t* test with Bonferroni correction. The error bars represent SEMs. Data underlying this figure can be found in S6 Data. GC, gustatory cortex; IOC, intraoral cannulae; LDA, linear discriminant analysis; LiCl, lithium chloride.

We therefore moved on to performing this direct test—2 such tests, in fact. First, we collected a new dataset using a modified experimental protocol in which we administered (and acquired spiking responses for *n* = 74 GC neurons to) tastes starting immediately after the injection of LiCl (**Fig 7A**). As already established, illness-related behaviors and changes in GC μ power emerge by 20 min after LiCl injection (**Figs 2 and 3**); we therefore hypothesized that the above-described coding changes would emerge within single ensembles at approximately this same time point—that coding in trials delivered before sickness onset (“Pre”) would differ from that in trials delivered after sickness onset (“Post”; **Fig 7A**).

We again used LDA to test whether and when taste response coding changed by testing the similarity of the first 5 “healthy” trials of each tastant to each subsequent taste delivery (binned; 5 trials/taste/bin). As expected, a repeated measures ANOVA performed between bins (bin = 6 min, approximately 3 trials/min) on taste responsive neurons (*n* = 56) revealed that the classifiability of tastes decreased significantly as rats became ill (*F*(4, 8) = 5.42, *p* = 0.02, *np*^2^ = 0.64), with this disruption becoming significant approximately 30 min into the session (**Fig 7B**, *ps* < 0.05). This result is a fairly good match for the above-described changes in GC μ power and illness-related behaviors (**Figs 2 and 3**), particularly given the necessarily small sample of trials. While one could speculate that this effect is driven simply by time (as opposed to illness), this explanation is rendered unlikely by previous results demonstrating that, in the absence of illness, within-session taste discriminability does not vary significantly across much longer time spans [26]. The far more likely explanation is that taste responses change following the onset of illness.

**Fig 8:**
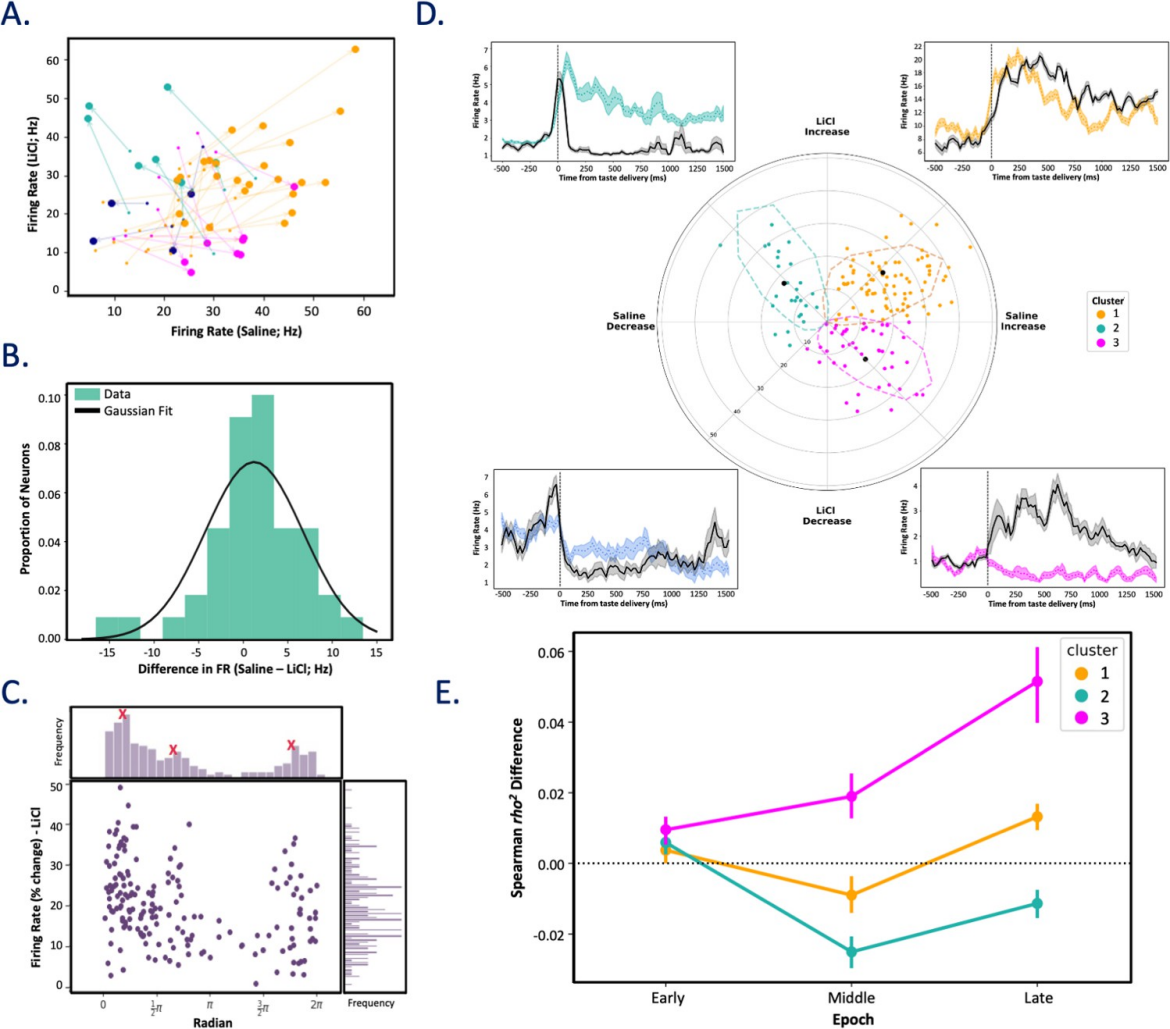
Neurons held across post-LiCl and post-saline tasting sessions produce distinct clusters of taste response profiles. **(A)** Firing rate trajectories for single neurons before (small circles) and after (large circles) taste delivery to the presentation of citric acid reveal condition-specific (sickness–y-axis, healthy–x-axis) taste response properties. Responses are colored with respect to angle of the response trajectory (0–90°, 90–180°, 180–270°, and 270–360° are yellow, teal, magenta, and blue, respectively), which highlights the distinct natures (excitatory, inhibitory) of responses in each condition. **(B)** A comparison of pre-stimulus activity suggests that post-injection firing rates did not differ between conditions: The peak of the distribution of saline-LiCl differences hovers around 0, and the distribution is fit well by a Gaussian (Kolmogorov–Smirnov test; *p* = 0.98); these facts suggest 0 difference (plus random noise). **(C)** A population response histogram (*n* = 44) held neurons depicting each neuron’s taste response (change from pre taste) as a function of vector direction in panel B confirms the presence of 3 unique peaks of vector direction (peaks indicated with red Xs). **(D)** A GMM was used to cluster cells based on response magnitude and trajectory. This analysis confirmed distinct cluster responses, revealed here in a polar plot (dashed lines denote 95% confidence interval of cluster classification with respect to black-filled centroids). PSTHs for representative neurons are seen adjacent to the detected clusters, indicating taste responses post-saline (black) and post-LiCl (colored, cluster specific). **(E)** Cluster 3 (magenta) neurons reliably and selectively experience a sickness-induced increase in palatability-relatedness in the late epoch (nonparametric 2-way repeated-measure ANOVA; *** *p* <0.001). Vertical lines represent SEMs. Data underlying this figure can be found in S7 Data. GMM, Gaussian mixture model; LiCl, lithium chloride; PSTH, peristimulus time histogram.

We went on to more closely examine the precise nature of these changes in the subsample of our recorded neurons that were held across sessions—neurons in which we were able to assay responses after both LiCl and saline injections (see Methods for criteria). For each of 44 held neurons, we first examined session-specific firing rates for the 500 ms before and after taste delivery (starting after the initial 200 ms, which, as expected, was shown to be devoid of taste information in paired *t* tests). Visual examination of the data suggests that pre-stimulus firing rates in these neurons were not affected by LiCl administration (note that the small dots, which plot pre-stimulus firing, form a cloud going up the diagonal of **Fig 8A**, which plots the data for 1 representative taste [CA]). A paired *t* test confirmed that the mean pre-stimulus activity did not differ between saline (*M* = 6.68, SD = 4.71) and LiCl (*M* = 5.46, SD = 5.02) sessions (t(43) = 1.46, *p* = 0.15), and a Kolmogorov–Smirnov test confirmed the lack of a condition-specific impact on baseline firing rates (D(44) = 0.045, *p* = 0.98; **Fig 8B**), revealing that saline-LiCl difference in pre-stimulus firing rates were normally distributed and centered around 0.

Further scrutiny of **Fig 8A**, meanwhile, suggests 3 distinct “types” of impact. Cluster analyses confirmed these appearances, revealing 3 distinct and discrete clusters of taste-response changes wrought by sickness (identified with distinct colors attached to neurons in each cluster): (1) in some neurons, taste responses were excitatory in saline sessions but inhibitory during LiCl sessions; (2) in others, the reverse was true; and (3) in a third group of neurons, responses were excitatory in both states, but these excitatory responses were generally less strong following LiCl injections. Ancillary analysis confirmed the presence of these clusters in the full dataset, showing 3 peaks in the angle of the **Fig 8A** vectors (**Fig 8C and 8D,** the latter with representative examples of each type, as well as of the few neurons that fail to cluster).

These 3 clusters of neurons, which were sorted without regard to the types of information within the taste response, nonetheless differed with regard to how illness-inducing LiCl impacted palatability-relatedness in the dynamics of the taste responses. A 3-way nonparametric mixed ANOVA run on the palatability content of all neurons represented in **Fig 8D** revealed a significant interaction between cluster, epoch, and condition, *F*(4, 478) = 4.80, *p* = 0.001, indicating that the relationship between cluster (whether the neurons were in cluster 1, 2, or 3) and the coding of palatability within epoch was significantly different across the saline and LiCl conditions. Specifically, the late epoch enhancement of palatability-related firing described earlier proved to be primarily a function of one group of neurons—those for which normally excitatory responses were turned into inhibitory responses by LiCl-induced illness (*F*(1, 130) = 33.47, *p* = 5.12 × 10^−8^; **Fig 8E**). This result both confirms the validity of the separation of neuron types and reveals that this separation is related to the impact of illness state on palatability processing.

## Discussion

Nutritional requirements, environmental conditions, and experience work together to guide consummatory behavior [2,11,13], in cooperation with an animal’s internal state (e.g., hunger, illness) [1]. The study of how physiological states impact perception is an important one, because this relationship intrinsically controls the animal’s likelihood of survival. The fact that neurons are embedded in networks, and that the states of such networks can be indexed in terms of EEG power (particularly in θ and μ frequency ranges [25,26,62]), motivated our decision to characterize changes in LFP activity within GC as an animal shifts from a healthy state into one of emesis, and to then relate this shift to changes in the dynamics of taste coding. This analysis reveals a modulation of μ power occurring approximately 15 min after LiCl injection—a result consistent with human [77] and rat data [78].

The finding that rearing durations, a measure of healthy exploratory behavior [2,70,71,78–80], drop at approximately the same time is consistent with decades of work showing that poisoning-related behaviors, and specifically those brought on by systemic nausea, emerge (depending on dose) at roughly 10 to 15 min post administration [2–4,78]. In our hands, the emergence of this LiCl-induced behavioral change closely mirrors the change in GC μ power induced by the same LiCl dose, implicating the process whereby an animal experiences illness in generation of these neural changes.

Of course, the comparable timing of GC LFP activity and the emergence of sickness (as indicated by behavioral changes) cannot exclude the possibility that changes in GC activity could conceivably reflect, not illness per se, but rather a direct effect of LiCl on GC neurons. While unlikely, this alternative explanation would persist as a possibility even if we had shown illness-related behaviors and GC changes in the same rats (we used separate groups because the intentional subtlety of the illness-related rearing changes, which allowed us to avoid the possibility of GC changes reflecting massive changes in oral fluid handling, cannot be observed in rats attached to the electrophysiology/IOC harness). To completely disapprove this hypothesis, an additional set of experiments would be required, in which we somehow block every pathway whereby visceral inputs carry information regarding LiCl-induced gastrointestinal distress and test whether this manipulation also blocks cortical changes. In the absence of the huge expenditure of time and resources required for these experiments, the best solution involved testing a particularly rigorous and risky prediction: Rather than just predicting that LiCl would change cortical activity, we specifically predicted that LiCl would change cortical activity at about the same latency that it caused illness (measured in rearing behavior). Given the success of this prediction (see Figs 2, 3, and 7), the most likely explanation (by far) is that the 2 are linked—that LiCl-induced illness is reflected in cortical activity.

The change in GC μ power is phasic, meaning that it is not simply the case that health connotes one μ amplitude and illness a different μ amplitude. This concept is not novel—while body states can be indexed in terms of LFP power fluctuations, behavioral states (e.g., sleep/wake) and muscle movements do not always correspond with a particular amplitude of field potential discharge [81] nor do significant changes in LFP power necessarily imply an observable change in body states [82]. Like many dynamical systems, the cortex experiences transient periods of instability at the time of state changes; we postulate that the observed transient impact of LiCl administration on GC μ power signals the onset of an illness state, and that the relaxation to a lower power regime shortly (approximately 7 min) thereafter nonetheless leaves the network changed in a way that impacts the processing of internal and external stimuli. In this, GC is akin to an automobile engine: Shifting from one gear to the next requires a brief transition from a steady state to a transient and then back to a steady state for optimal efficiency [83,84].

In addition to altering μ power, LiCl also significantly altered GC taste responses. This fact dovetails nicely with data presented by Arieli and colleagues [85], who examined how GC activity changes following CTA learning, identifying 2 types of impact resulting from the pairing of taste with LiCl-induced illness—an “immediate” impact of LiCl administration on taste responses in GC single neurons and a “delayed” impact on GC ensemble population dynamics in taste response. Arieli and colleagues hypothesized that the late impact is driven by CTA formation, and that the immediate impact is likely caused by the illness state induced by LiCl injection. Our current findings are consistent with theirs.

We went on to show that the impact of LiCl administration on taste coding is epoch dependent. The drug disrupted identity coding while (somewhat incongruously) enhancing the palatability-relatedness of the responses, with the latter effect localized to the oft-described late epoch of the responses [30–32]. We also observed a small but significant illness-related increase in palatability coding in the early epoch. While an explanation for this unexpected result awaits further experimentation, we would speculate, based on work showing that expectation of stimulus availability can reduce that latency of gustatory coding (effectively eliminating the 200 ms non-chemosensory period observed in our work, see [38,46–50]), that illness might similarly enhance the “readiness” of cortex and reduce the latency of taste-specific coding; since illness biases GC toward palatability-related coding (see below), it could very well be palatability coding that appears early when the animal is ill.

These findings may at first blush appear to be in conflict with previous studies examining behavioral changes following LiCl-induced illness [86–88], which suggested that sickness has no effect on palatability (e.g., as measured via evaluation of taste reactivity to a single taste [86]). In fact, however, our findings regarding sickness polarizing palatability processing are largely consistent with these results, in that they do not suggest wholesale changes in palatability of individual tastes, or even changes in the order of preference. Rather, we argue that illness simplifies taste coding, making aversive tastes more similarly aversive and palatable tastes more similarly palatable.

The results described in Figs 4–6 suggest, but do not prove, that single neurons change their coding as a function of illness. It is always possible that neurons coding taste identity and palatability during illness represent a distinct set of neurons from those coding these properties in healthy rats, and that the differences in overall sample responses reflected the addition of this new set of “illness-only” responses. In the first test of the hypothesis that illness changes the processing of taste stimuli in individual neural ensembles, we showed that taste discriminability is significantly changed as an animal enters the emetic state. And since the small number of trials available in this analysis rendered it impossible to reliably compare palatability correlations, we performed a second analysis in which we leveraged our ability to hold a subset of our single neurons across both testing sessions. This analysis revealed that LiCl-induced illness enhances palatability-relatedness in an epoch-specific manner but went beyond this to reveal 3 distinct subpopulations of illness-related response profiles.

We cannot, as of yet, provide a definitive explanation of this functional dissociation of GC neurons, but we can speculate as to several mechanisms that could explain our results. The most parsimonious of these explanations might be that the clusters come from either functionally or physiologically distinct populations of cells which in turn code specific features about the state of the animal (and thus responses to external stimuli) in unique, yet beneficial ways. However, cross-correlational analysis (methods adapted from Li and colleagues [54]) failed to reveal differences in the strength of the functional connectivity between (nor within) the individual clusters, which would have been expected with such an explanation [54]. Furthermore, analysis of wave shapes and basal firing rates failed to reveal groups to be made up of different percentages of putative pyramidal and interneurons. While it is always risky to reach conclusions on the basis of a null result, particularly in small datasets, we find it unlikely that these clusters represent distinct types of GC neurons.

An alternative explanation for the observed differences in palatability coding across these putative clusters has to do with the possible sources of input to these neurons. It has recently been shown that specific area postrema neuron types are responsible for providing illness-related information to brain regions [18], and that these influences may reach GC by way of either the nucleus of the solitary tract [89] or other regions (e.g., amygdala) known to be impacted by the physiological state induced by LiCl [90]. It is possible that the palatability-coding differences between GC clusters are a downstream result of cell type–specific projections. Future work will examine this possibility, incorporating the use of video recordings of oral-facial reactivity to establish the link more concretely between these putative clusters and their behavioral relevancy in consummatory behaviors.

Regardless of the answers to these questions, the work presented here demonstrates that illness, or at least that caused by LiCl, impacts cortical function in a far more refined manner than via a simple wholesale disruption of activity. The effects of illness on GC activity are complex, but this complexity seems linked to an animal’s need, when ill, to simply tell good from bad. In fact, this is not the first case we have found a manipulation that biased GC response toward stimulus palatability. Fontanini and Katz [26] also found that when the behavioral state of an animal changed from a task-oriented to a disengaged (or inattentive) state, GC response became less discriminative between palatability-similar tastes but more distinct for tastes with opposite valences. The similarity in impact of illness and engagement states provides further support for the general finding that GC is involved in the making and putting into action of decisions related to palatability [32,91]: While the most basic circuit involved in these decisions is found in the brainstem [73,74,92], the taste system is very much like other vertebrate and invertebrate sensorimotor systems [92]—in situ, top-down modulation plays a major role in determining behavior. Here, GC plays that role, and this is possibly why body states are reflected in GC as well.

## Supporting information

S1 Data S1_Data.xlsx includes the data underlying Fig 2. (XLSX)

S2 Data S2_Data.xlsx includes the data underlying Fig 3. (XLSX)

S3 Data S3_Data.xlsx includes the data underlying Fig 4. (XLSX)

S4 Data S4_Data.xlsx includes the data underlying Fig 5. (XLSX)

S5 Data S5_Data.xlsx includes the data underlying Fig 6. (XLSX)

S6 Data S6_Data.xlsx includes the data underlying Fig 7. (XLSX)

S7 Data S7_Data.xlsx includes the data underlying Fig 8. (XLSX)

## Abbreviations

CTA: conditioned taste aversion
GC: gustatory cortex
GMM: Gaussian mixture model
IOC: intraoral cannulae
IP: intraperitoneal
LDA: linear discriminant analysis
LFP: local field potential
LiCl: lithium chloride
PPI: pure palatability index
RD: response difference

## References

[1] Livneh Y, Sugden AU, Madara JC, Essner RA, Flores VI, Sugden LA, et al. Estimation of current and future physiological states in insular cortex. Neuron. 2020;105(6):1094–111. e10. doi: 10.1016/j.neuron.2019.12.027 31955944 PMC7083695

[2] Parker LA. Nonconsummatory and consummatory behavioral CRs elicited by lithium-and amphetamine-paired flavors. Learn Motiv. 1982;13(3):281–303.

[3] Nachman M. Learned aversion to the taste of lithium chloride and generalization to other salts. J Comp Physiol Psychol. 1963;56(2):343. 13937024 10.1037/h0046484

[4] Nachman M, Ashe JH. Learned taste aversions in rats as a function of dosage, concentration, and route of administration of LiCl. Physiol Behav. 1973;10(1):73–78. doi: 10.1016/0031-9384(73)90089-9 4697023

[5] Löfhede J, Thordstein M, Löfgren N, Flisberg A, Rosa-Zurera M, Kjellmer I, et al. Automatic classification of background EEG activity in healthy and sick neonates. J Neural Eng. 2010;7(1):016007. doi: 10.1088/1741-2560/7/1/016007 20075506

[6] Li M, Lu B-L, editors. Emotion classification based on gamma-band EEG. Engineering in Medicine and Biology Society, 2009 EMBC 2009 Annual International Conference of the IEEE; 2009: IEEE. 10.1109/IEMBS.2009.5334139 19964505

[7] Okonogi T, Nakayama R, Sasaki T, Ikegaya Y. Characterization of Peripheral Activity States and Cortical Local Field Potentials of Mice in an Elevated Plus Maze Test. Front Behav Neurosci. 2018;12:62. doi: 10.3389/fnbeh.2018.00062 29666572 PMC5891585

[8] Kelly RC, Smith MA, Kass RE, Lee TS. Local field potentials indicate network state and account for neuronal response variability. J Comput Neurosci. 2010;29(3):567–579. doi: 10.1007/s10827-009-0208-9 20094906 PMC3604740

[9] Aubert A. Sickness and behaviour in animals: a motivational perspective. Neurosci Biobehav Rev. 1999;23(7):1029–1036. doi: 10.1016/s0149-7634(99)00034-2 10580315

[10] Aubert A, Dantzer R. The taste of sickness: lipopolysaccharide-induced finickiness in rats. Physiol Behav. 2005;84(3):437–444. doi: 10.1016/j.physbeh.2005.01.006 15763581

[11] Provenza FD. Postingestive feedback as an elementary determinant of food preference and intake in ruminants. Rangel Ecol Manag/J Range Manag Archives. 1995;48(1):2–17.

[12] Garcia J, Hankins WG, Rusiniak KW. Behavioral regulation of the milieu interne in man and rat. Science. 1974;185(4154):824–831. doi: 10.1126/science.185.4154.824 11785521

[13] Flores VL, Parmet T, Mukherjee N, Nelson S, Katz DB, Levitan D. The role of the gustatory cortex in incidental experience-evoked enhancement of later taste learning. Learn Mem. 2018;25(11):587–600. doi: 10.1101/lm.048181.118 30322892 PMC6191014

[14] Lin J-Y, Arthurs J, Reilly S. Conditioned taste aversion, drugs of abuse and palatability. Neurosci Biobehav Rev. 2014;45:28–45. doi: 10.1016/j.neubiorev.2014.05.001 24813806 PMC4134772

[15] Cloutier CJ, Kavaliers M, Ossenkopp K-P. Rodent sex differences in disgust behaviors (anticipatory nausea) conditioned to a context associated with the effects of the toxin LiCl: Inhibition of conditioning following immune stimulation with lipopolysaccharide. Pharmacol Biochem Behav. 2017;152:4–12. doi: 10.1016/j.pbb.2016.08.006 27566289

[16] Cloutier CJ, Kavaliers M, Ossenkopp K-P. Lipopolysaccharide inhibits the simultaneous establishment of LiCl-induced anticipatory nausea and intravascularly conditioned taste avoidance in the rat. Behav Brain Res. 2012;232(1):278–286. doi: 10.1016/j.bbr.2012.04.021 22537776

[17] Cloutier CJ, Rodowa M-S, Cross-Mellor SK, Chan MY, Kavaliers M, Ossenkopp K-P. Inhibition of LiCl-induced conditioning of anticipatory nausea in rats following immune system stimulation: comparing the immunogens lipopolysaccharide, muramyl dipeptide, and polyinosinic: polycytidylic acid. Physiol Behav. 2012;106(2):243–251. doi: 10.1016/j.physbeh.2012.02.005 22342813

[18] Zhang C, Kaye JA, Cai Z, Wang Y, Prescott SL, Liberles SD. Area postrema cell types that mediate nausea-associated behaviors. Neuron. 2021;109(3):461–72. e5. doi: 10.1016/j.neuron.2020.11.010 33278342 PMC7864887

[19] Nachman M, Hartley PL. Role of illness in producing learned taste aversions in rats: A comparison of several rodenticides. J Comp Physiol Psychol. 1975;89(9):1010. doi: 10.1037/h0077189 1238440

[20] Ching S, Brown EN. Modeling the dynamical effects of anesthesia on brain circuits. Curr Opin Neurobiol. 2014;25:116–122. doi: 10.1016/j.conb.2013.12.011 24457211 PMC4181389

[21] Cimenser A, Purdon PL, Pierce ET, Walsh JL, Salazar-Gomez AF, Harrell PG, et al. Tracking brain states under general anesthesia by using global coherence analysis. Proc Natl Acad Sci U S A. 2011;108(21):8832–8837. doi: 10.1073/pnas.1017041108 21555565 PMC3102391

[22] Olcese U, Oude Lohuis MN, Pennartz C. Sensory processing across conscious and nonconscious brain states: from single neurons to distributed networks for inferential representation. Front Syst Neurosci. 2018;12:49. doi: 10.3389/fnsys.2018.00049 30364373 PMC6193318

[23] Lee S-H, Dan Y. Neuromodulation of brain states. Neuron. 2012;76(1):209–222. doi: 10.1016/j.neuron.2012.09.012 23040816 PMC3579548

[24] Canolty RT, Ganguly K, Carmena JM. Task-dependent changes in cross-level coupling between single neurons and oscillatory activity in multiscale networks. PLoS Comput Biol. 2012;8(12):e1002809. doi: 10.1371/journal.pcbi.1002809 23284276 PMC3527280

[25] Fontanini A, Katz DB. 7 to 12 Hz activity in rat gustatory cortex reflects disengagement from a fluid self-administration task. J Neurophysiol. 2005;93(5):2832–2840. doi: 10.1152/jn.01035.2004 15574797

[26] Fontanini A, Katz DB. State-dependent modulation of time-varying gustatory responses. J Neurophysiol. 2006;96(6):3183–3193. doi: 10.1152/jn.00804.2006 16928791

[27] Towers AE, York JM, Baynard T, Gainey SJ, Freund GG. Mouse testing methods in psychoneuroimmunology 2.0: measuring behavioral responses. Psychoneuroimmunology: Springer. 2018:221–258. doi: 10.1007/978-1-4939-7828-1_13 29705851

[28] Kent S, Bluthé R-M, Kelley KW, Dantzer R. Sickness behavior as a new target for drug development. Trends Pharmacol Sci. 1992;13:24–28. doi: 10.1016/0165-6147(92)90012-u 1542935

[29] Mukherjee N, Wachutka J, Katz DB. Python meets systems neuroscience: affordable, scalable and open-source electrophysiology in awake, behaving rodents. 2017.

[30] Katz D, Simon S, Nicolelis M. Electrophysiological studies of gustation in awake rats. Methods and frontiers in the chemical senses. Simon SA, Nicolelis MAL, editors. 2001:339–57.

[31] Katz DB, Simon S, Nicolelis MA. Dynamic and multimodal responses of gustatory cortical neurons in awake rats. J Neurosci. 2001;21(12):4478–4489. doi: 10.1523/JNEUROSCI.21-12-04478.2001 11404435 PMC6762775

[32] Sadacca BF, Mukherjee N, Vladusich T, Li JX, Katz DB, Miller P. The behavioral relevance of cortical neural ensemble responses emerges suddenly. J Neurosci. 2016;36(3):655–669. doi: 10.1523/JNEUROSCI.2265-15.2016 26791199 PMC4719008

[33] Grossman SE, Fontanini A, Wieskopf JS, Katz DB. Learning-related plasticity of temporal coding in simultaneously recorded amygdala–cortical ensembles. J Neurosci. 2008;28(11):2864–2873. doi: 10.1523/JNEUROSCI.4063-07.2008 18337417 PMC6670663

[34] Levitan D, Lin J-Y, Wachutka J, Mukherjee N, Nelson SB, Katz DB. Single and population coding of taste in the gustatory cortex of awake mice. J Neurophysiol. 2019;122(4):1342–1356. doi: 10.1152/jn.00357.2019 31339800 PMC6843090

[35] Moran A, Katz DB. Sensory cortical population dynamics uniquely track behavior across learning and extinction. J Neurosci. 2014;34(4):1248–1257. doi: 10.1523/JNEUROSCI.3331-13.2014 24453316 PMC3898286

[36] Sadacca BF, Rothwax JT, Katz DB. Sodium concentration coding gives way to evaluative coding in cortex and amygdala. J Neurosci. 2012;32(29):9999–10011. doi: 10.1523/JNEUROSCI.6059-11.2012 22815514 PMC3432403

[37] Spector AC, Grill HJ. Differences in the taste quality of maltose and sucrose in rats: issues involving the generalization of conditioned taste aversions. Chem Senses. 1988;13(1):95–113.

[38] Samuelsen CL, Gardner MP, Fontanini A. Effects of cue-triggered expectation on cortical processing of taste. Neuron. 2012;74(2):410–422. doi: 10.1016/j.neuron.2012.02.031 22542192 PMC3340578

[39] Smith JC. Radiation: Its detection and its effects on taste preferences. Prog Physiol Psychol. 1971;4:53–118.

[40] Cross-Mellor SK, Foley KA, Parker LA, Ossenkopp K-P. Lipopolysaccharide dose dependently impairs rapid toxin (LiCl)-induced gustatory conditioning: a taste reactivity examination of the conditioned taste aversion. Brain Behav Immun. 2009;23(2):204–216. doi: 10.1016/j.bbi.2008.09.006 18835436

[41] Tort AB, Fontanini A, Kramer MA, Jones-Lush LM, Kopell NJ, Katz DB. Cortical networks produce three distinct 7–12 Hz rhythms during single sensory responses in the awake rat. J Neurosci. 2010;30(12):4315–4324. doi: 10.1523/JNEUROSCI.6051-09.2010 20335467 PMC3318968

[42] Tort AB, Komorowski R, Eichenbaum H, Kopell N. Measuring phase-amplitude coupling between neuronal oscillations of different frequencies. J Neurophysiol. 2010;104(2):1195–1210. doi: 10.1152/jn.00106.2010 20463205 PMC2941206

[43] Thomson DJ. Jackknife error estimates for spectra, coherences, and transfer functions. Advances in Spectral Analysis and Array Processing. 1991. p. 58–113.

[44] Salvatier J, Wiecki TV, Fonnesbeck C. Probabilistic programming in Python using PyMC3. PeerJ Comput Sci. 2016;2:e55. 10.7717/peerj-cs.1516 PMC10495961 37705656

[45] Jones LM, Fontanini A, Sadacca BF, Miller P, Katz DB. Natural stimuli evoke dynamic sequences of states in sensory cortical ensembles. Proc Natl Acad Sci U S A. 2007;104(47):18772–18777. doi: 10.1073/pnas.0705546104 18000059 PMC2141852

[46] Gutierrez R, Simon SA, Nicolelis MA. Licking-induced synchrony in the taste–reward circuit improves cue discrimination during learning. J Neurosci. 2010;30(1):287–303. doi: 10.1523/JNEUROSCI.0855-09.2010 20053910 PMC2831544

[47] Stapleton JR, Lavine ML, Wolpert RL, Nicolelis MA, Simon SA. Rapid taste responses in the gustatory cortex during licking. J Neurosci. 2006;26(15):4126–4138. doi: 10.1523/JNEUROSCI.0092-06.2006 16611830 PMC6673900

[48] Graham DM, Sun C, Hill DL. Temporal signatures of taste quality driven by active sensing. J Neurosci. 2014;34(22):7398–7411. doi: 10.1523/JNEUROSCI.0213-14.2014 24872546 PMC4035510

[49] Bouaichi CG, Vincis R. Cortical processing of chemosensory and hedonic features of taste in active licking mice. J Neurophysiol. 2020;123(5):1995–2009. doi: 10.1152/jn.00069.2020 32319839 PMC7444915

[50] Dikecligil GN, Graham DM, Park IM, Fontanini A. Layer-and cell Type-Specific response properties of gustatory cortex neurons in awake mice. J Neurosci. 2020;40(50):9676–9691. doi: 10.1523/JNEUROSCI.1579-19.2020 33172981 PMC7726536

[51] Li JX, Maier JX, Reid EE, Katz DB. Sensory cortical activity is related to the selection of a rhythmic motor action pattern. J Neurosci. 2016;36(20):5596–5607. doi: 10.1523/JNEUROSCI.3949-15.2016 27194338 PMC4871991

[52] Nicolelis MA, Lin RC, Chapin JK. Neonatal whisker removal reduces the discrimination of tactile stimuli by thalamic ensembles in adult rats. J Neurophysiol. 1997;78(3):1691–1706. doi: 10.1152/jn.1997.78.3.1691 9310453

[53] Piette CE, Baez-Santiago MA, Reid EE, Katz DB, Moran A. Inactivation of basolateral amygdala specifically eliminates palatability-related information in cortical sensory responses. J Neurosci. 2012;32(29):9981–9991. doi: 10.1523/JNEUROSCI.0669-12.2012 22815512 PMC3432404

[54] Li JX, Yoshida T, Monk KJ, Katz DB. Lateral hypothalamus contains two types of palatability-related taste responses with distinct dynamics. J Neurosci. 2013;33(22):9462–9473. doi: 10.1523/JNEUROSCI.3935-12.2013 23719813 PMC3960458

[55] Fontanini A, Grossman SE, Figueroa JA, Katz DB. Distinct subtypes of basolateral amygdala taste neurons reflect palatability and reward. J Neurosci. 2009;29(8):2486–2495. doi: 10.1523/JNEUROSCI.3898-08.2009 19244523 PMC2668607

[56] Nicolelis MA, Dimitrov D, Carmena JM, Crist R, Lehew G, Kralik JD, et al. Chronic, multisite, multielectrode recordings in macaque monkeys. Proc Natl Acad Sci U S A. 2003;100(19):11041–11046. doi: 10.1073/pnas.1934665100 12960378 PMC196923

[57] Herry C, Ciocchi S, Senn V, Demmou L, Müller C, Lüthi A. Switching on and off fear by distinct neuronal circuits. Nature. 2008;454(7204):600–606. doi: 10.1038/nature07166 18615015

[58] Athey TL, Vogelstein JT. AutoGMM: Automatic Gaussian Mixture Modeling in Python. arXiv preprint arXiv:190902688. 2019.

[59] Mukamel EA, Pirondini E, Babadi B, Wong KFK, Pierce ET, Harrell PG, et al. A transition in brain state during propofol-induced unconsciousness. J Neurosci. 2014;34(3):839–845. doi: 10.1523/JNEUROSCI.5813-12.2014 24431442 PMC3891963

[60] Abásolo D, Simons S, Morgado da Silva R, Tononi G, Vyazovskiy VV. Lempel-Ziv complexity of cortical activity during sleep and waking in rats. J Neurophysiol. 2015;113(7):2742–2752. doi: 10.1152/jn.00575.2014 25717159 PMC4416627

[61] Manning JR, Jacobs J, Fried I, Kahana MJ. Broadband shifts in local field potential power spectra are correlated with single-neuron spiking in humans. J Neurosci. 2009;29(43):13613–13620. doi: 10.1523/JNEUROSCI.2041-09.2009 19864573 PMC3001247

[62] Fontanini A, Katz DB. Behavioral states, network states, and sensory response variability. J Neurophysiol. 2008;100(3):1160–1168. doi: 10.1152/jn.90592.2008 18614753 PMC2544460

[63] Vijayan S, Ching S, Purdon PL, Brown EN, Kopell NJ. Thalamocortical mechanisms for the anteriorization of alpha rhythms during propofol-induced unconsciousness. J Neurosci. 2013;33(27):11070–11075. doi: 10.1523/JNEUROSCI.5670-12.2013 23825412 PMC3718379

[64] Waldert S, Lemon RN, Kraskov A. Influence of spiking activity on cortical local field potentials. J Physiol. 2013;591(21):5291–5303. doi: 10.1113/jphysiol.2013.258228 23981719 PMC3936368

[65] Pachitariu M, Lyamzin DR, Sahani M, Lesica NA. State-dependent population coding in primary auditory cortex. J Neurosci. 2015;35(5):2058–2073. doi: 10.1523/JNEUROSCI.3318-14.2015 25653363 PMC4315834

[66] Zhou M, Liang F, Xiong XR, Li L, Li H, Xiao Z, et al. Scaling down of balanced excitation and inhibition by active behavioral states in auditory cortex. Nat Neurosci. 2014;17(6):841–850. doi: 10.1038/nn.3701 24747575 PMC4108079

[67] Tan AY, Chen Y, Scholl B, Seidemann E, Priebe NJ. Sensory stimulation shifts visual cortex from synchronous to asynchronous states. Nature. 2014;509(7499):226–229. doi: 10.1038/nature13159 24695217 PMC4067243

[68] Zucca S, Pasquale V, de Leon Roig PL, Panzeri S, Fellin T. Thalamic drive of cortical parvalbumin-positive interneurons during down states in anesthetized mice. Curr Biol. 2019;29(9):1481–90. e6. doi: 10.1016/j.cub.2019.04.007 31031117 PMC6509281

[69] Parker LA, Kwiatkowska M, Burton P, Mechoulam R. Effect of cannabinoids on lithium-induced vomiting in the Suncus murinus (house musk shrew). Psychopharmacology. 2004;171(2):156–161. doi: 10.1007/s00213-003-1571-2 13680081

[70] Tomasiewicz HC, Mague SD, Cohen BM, Carlezon WA Jr. Behavioral effects of short-term administration of lithium and valproic acid in rats. Brain Res. 2006;1093(1):83–94. doi: 10.1016/j.brainres.2006.03.102 16687130

[71] López M, Dwyer DM, Gasalla P. Conditioned hedonic responses elicited by contextual cues paired with nausea or with internal pain. Behav Neurosci. 2019;133(1):86. doi: 10.1037/bne0000291 30589272

[72] Smith D. Learned aversion and rearing movement in rats given LiCl, PbCl 2 or NaCl. Experientia. 1978;34(9):1200–1201. doi: 10.1007/BF01922956 720522

[73] Grill HJ, Norgren R. The taste reactivity test. II. Mimetic responses to gustatory stimuli in chronic thalamic and chronic decerebrate rats. Brain Res. 1978;143(2):281–297. doi: 10.1016/0006-8993(78)90569-3 630410

[74] Grill HJ, Norgren R. The taste reactivity test. I. Mimetic responses to gustatory stimuli in neurologically normal rats. Brain Res. 1978;143(2):263–279. doi: 10.1016/0006-8993(78)90568-1 630409

[75] Shadlen MN, Newsome WT. Noise, neural codes and cortical organization. Curr Opin Neurobiol. 1994;4(4):569–579. doi: 10.1016/0959-4388(94)90059-0 7812147

[76] Shadlen MN, Newsome WT. The variable discharge of cortical neurons: implications for connectivity, computation, and information coding. J Neurosci. 1998;18(10):3870–3896. doi: 10.1523/JNEUROSCI.18-10-03870.1998 9570816 PMC6793166

[77] Chen Y-C, Duann J-R, Chuang S-W, Lin C-L, Ko L-W, Jung T-P, et al. Spatial and temporal EEG dynamics of motion sickness. NeuroImage. 2010;49(3):2862–2870. doi: 10.1016/j.neuroimage.2009.10.005 19833217

[78] Aguilar-Rivera M, Kim S, Coleman TP, Maldonado PE, Torrealba F. Interoceptive insular cortex participates in sensory processing of gastrointestinal malaise and associated behaviors. Sci Rep. 2020;10(1):1–12. 33303809 10.1038/s41598-020-78200-w PMC7730439

[79] Alves R, de Carvalho JGB, Benedito MAC. High and low rearing subgroups of rats selected in the open field differ in the activity of K+-stimulated p-nitrophenylphosphatase in the hippocampus. Brain Res. 2005;1058(1–2):178–182. doi: 10.1016/j.brainres.2005.08.005 16153614

[80] Rosana A, Campana MA, V. High-and low-rearing rats differ in the brain excitability controlled by the allosteric benzodiazepine site in the GABA A receptor. Behav Brain Sci. 2012;2012.

[81] Aston-Jones G, Bloom F. Activity of norepinephrine-containing locus coeruleus neurons in behaving rats anticipates fluctuations in the sleep-waking cycle. J Neurosci. 1981;1(8):876–886. doi: 10.1523/JNEUROSCI.01-08-00876.1981 7346592 PMC6564235

[82] Rojas-Líbano D, Frederick DE, Egaña JI, Kay LM. The olfactory bulb theta rhythm follows all frequencies of diaphragmatic respiration in the freely behaving rat. Front Behav Neurosci. 2014;8:214. doi: 10.3389/fnbeh.2014.00214 24966821 PMC4053074

[83] Oglieve CJ, Mohammadpour M, Rahnejat H. Optimisation of the vehicle transmission and the gear-shifting strategy for the minimum fuel consumption and the minimum nitrogen oxide emissions. Proc Inst Mech Eng D. 2017;231(7):883–899.

[84] Van Horn C. Steady state and transient efficiencies of a four cylinder direct injection diesel engine for implementation in a hybrid electric vehicle. University of Akron; 2006.

[85] Arieli E, Younis N, Moran A. Distinct progressions of neuronal activity changes underlie the formation and consolidation of a gustatory associative memory. J Neurosci. 2022;42(5):909–921. doi: 10.1523/JNEUROSCI.1599-21.2021 34916257 PMC8808731

[86] Spector AC, Breslin P, Grill HJ. Taste reactivity as a dependent measure of the rapid formation of conditioned taste aversion: a tool for the neural analysis of taste-visceral associations. Behav Neurosci. 1988;102(6):942. doi: 10.1037//0735-7044.102.6.942 2850815

[87] Baird J-P, St John SJ, Nguyen EA-N. Temporal and qualitative dynamics of conditioned taste aversion processing: combined generalization testing and licking microstructure analysis. Behav Neurosci. 2005;119(4):983. doi: 10.1037/0735-7044.119.4.983 16187827

[88] Eckel LA, Ossenkopp K-P. Area postrema mediates the formation of rapid, conditioned palatability shifts in lithium-treated rats. Behav Neurosci. 1996;110(1):202. 8652067

[89] Shapiro RE, Miselis RR. The central neural connections of the area postrema of the rat. J Comp Neurol. 1985;234(3):344–364. doi: 10.1002/cne.902340306 3988989

[90] Spencer CM, Eckel LA, Nardos R, Houpt TA. Area postrema lesions attenuate LiCl-induced c-Fos expression correlated with conditioned taste aversion learning. Physiol Behav. 2012;105(2):151–160. doi: 10.1016/j.physbeh.2011.08.022 21889521 PMC3225712

[91] Mukherjee N, Wachutka J, Katz DB, editors. Dynamical Structure of Cortical Taste Responses Revealed by Precisely-timed Optogenetic Perturbation. CHEMICAL SENSES; 2018: OXFORD UNIV PRESS GREAT CLARENDON ST, OXFORD OX2 6DP, ENGLAND.

[92] Geran LC, Travers SP. Single neurons in the nucleus of the solitary tract respond selectively to bitter taste stimuli. J Neurophysiol. 2006;96(5):2513–2527. doi: 10.1152/jn.00607.2006 16899635

