# Supplementary material for: LiCl-induced sickness modulates rat gustatory cortical responses
Source: PLoS Biol. 2022 Jul 25;20(7):e3001537. doi: 10.1371/journal.pbio.3001537 (PMC9352195; doi:10.1371/journal.pbio.3001537)
Supplement: Attachment — Submitted filename: PLOS Authors response_Final.docx [file pbio.3001537.s008.docx]

We are grateful to the reviewers for the generosity of their critical comments, and to the editors for inviting this extensively revised manuscript. Below, we describe our responses to each specific criticism, including our descriptions (and locations) of the changes made (underlined).

**Reviewer 1 (R1)**

**R1**: *“As far as I could tell, only the section titled "Illness-related changes in taste coding occur in single neurons", and its corresponding figure (Fig. 7), lists the number of animals (n=3), number of total recorded neurons (n=74), and the subset of taste responsive neurons (n=56) used for the analyses. It is entirely unclear to me what populations of single-units are being compared in the section titled "Illness causes taste responses to resemble a good-bad distinction" (Fig. 4, Fig. 5, and Fig. 6). Are these populations from the same rats, but different days? If so, are they recorded at the same depth? Are the analyses performed across all neurons, only taste responsive neurons, or only the taste responsive neurons with stable waveforms across days (putative single-neurons)? What constitutes a "change in firing rate"? Is this a neuron by neuron comparison in taste-evoked activity between LiCl- and Saline-sessions? Or is this a comparison between all recorded neurons from the different sessions? Is baseline firing rate taken into account, because a 5Hz change in activity may impact the system more for a neuron with a 5hz baseline than one with a 50Hz baseline. The lack of specific information impedes a clear understanding of the results and mars the author's interpretations.*

**Response**: We apologize for the confusing lack of information. In the revision, we have taken several steps to clarify what data were used in what analysis. We have added animal and neuron numbers to each figure panel, generated a table (now included in the Methods section) compiling data collected, and modified the text itself in multiple places, to highlight the fact that **the same experimental data (all neurons recorded for the main dataset) were used for Figures 2, 4, 5, & 6** (additional control data was also used in Figure 2); Figure 7 involved collection of a new dataset (Figure 7B focused on taste responsive neurons from this new sample), and Figure 8 used all neurons that were held across sessions from the main data set. We have also endeavored to clear up any remaining confusion as to how the terms “taste-specificity” and “firing rate change” were statistically defined (page 8).

**R1:** *“Relatedly, the presentation of the data would be significantly enhanced by the addition of representative examples to the figures describing the single-unit activity (i.e. Fig. 4, Fig. 6) and especially the figures showing changes in single-unit activity within a session (Fig. 7 and Fig. 8).”*

**Response**: Representative examples have been added to figures 4 and 8. Visualizing the phenomena described in Figures 6 & 7 using representative examples proved difficult and messy, and we’d therefore prefer to leave these figures as is in this regard; in particular, a representative example for Figure 7 would involve multiple panels with 4 PSTHs on each, and an attempt to discern that pairs of PSTHs from later in the session are on average slightly less distinctive than pairs of PSTHs from earlier. We hope that this suffices; if R1 and the editors feel that it is not, we will endeavor to comply.

**R1:** *“Would the authors please explain why the first 300ms of the taste evoked activity is removed for some analysis (i.e. directionality), but then used as a key component of the analyses of taste epochs. The timeframe of 300ms seems questionable, given the many studies from this group showing that taste identity begins to emerge ~200ms after stimulus delivery (Katz et al., 2001; Fontanini and Katz, 2006; Sadacca et al., 2012; Sadacca et al., 2016). What is the justification for ignoring data for one analyses, but including it for others? Does including the data eliminate the significant difference in the directionality analyses?”*

**Response**: R1 correctly notes that we were neither clear nor consistent enough in the original submission. The work done in our lab has repeatedly confirmed that the first 200-300 msec of GC neurons’ responses to taste delivery contain no chemosensory-specific content (but see our response to Reviewer #2 below). For that reason, we now typically restrict our analyses of these responses to the later epochs. For the revision, we have normalized our approach, so that all analyses look at the same response periods. Note that while this has required minor re-analysis in some cases, these re-analyses had no impact on our conclusions.

**R1*:*** *“Can the authors provide any evidence that the rats in the electrophysiological group experienced malaise? Especially since only 5 of the 7 exhibited a significant change in LFP? For example, LiCl-induced malaise can evoke a number of aversive oromotor and somatic responses (Spector et al., 1988), including gape, chin rub, head shake, and forelimb flail. Did either group (ephys. or behavior) perform more of these behaviors after LiCl-induced malise compared to Saline controls?”*

**Response**: We very purposefully wanted to avoid making our rats sick enough to engage in such obviously “illness-related” behaviors, which would have confounded our interpretation of the electrophysiological results (because some of those behaviors could directly alter neural firing in response to tastes). We accepted as a risk of this approach that some rats might simply fail to become ill, but considered this risk worthwhile because: 1) it would only render our results more conservative; and 2) several publications have suggested that illness can be detected at LiCl concentrations that are “sub-threshold” for the behaviors mentioned by R1. This logic, complete with these references, is now made explicit in the revision (pg 7).

**R1:** *“For the analyses represented by Figure 8, the authors say "pre-stimulus firing rates fall generally along the diagonal, demonstrating that spontaneous firing rates do not differ strongly between condition". Individual variability aside, do the spontaneous firing rates of putative single neurons (as a population) differ between the LiCl- and Saline-sessions? Statistical confirmation would further support the stability of the population across sessions.”*

**Response**: For the revision, we have added multiple analyses that provide convergent statistical confirmation of our (somewhat surprising) finding that illness has little impact on firing rates (other than those driven by taste administration) in GC single neurons (pgs 13-14 and Figure 8B). Most notably, these analyses show that: 1) neither mean baseline firing rates nor the between-neuron variability in those rates differed significantly between LiCl and saline sessions; and that 2) the distribution of LiCl-saline differences in firing rates prior to taste delivery is indistinguishable from a gaussian distribution centered on zero.

**R1:** *“The authors should take care when describing activity suppressed by taste delivery as "inhibitory". I have received this same criticism in the past because some readers wrongly assume that the authors are suggesting that these neurons are themselves inhibitory.”*

**Response**: We are grateful to R1 for pointing this out. For the revision, we have added text to the Methods section (pg 8) that concretely defines our use of the term “inhibitory responses” (i.e., responses in which taste delivery drives firing rates down to significantly below baseline) and specifically notes that “we make no claims regarding whether or not “inhibitory responses” are caused by direct inhibitory influence on these neurons; nor do we intend to imply that these are responses found in inhibitory interneurons.”

**R1:** *“The manuscript would benefit from a spelling and grammar check, as there were a number of typos throughout the article.”*

**Response**: Done!

**Reviewer 2 (R2)**

**R2**: *“It would be helpful to know some more information about the recording strategy to better interpret the data presented in figures 4-6. That is, were the electrodes lowered after each NaCl and LiCl recording sessions to record new ensembles of GC neurons? …[w]hile I do understand the challenges of an experimental design in which the same neurons are recorded (and held) during and after NaCl and LiCl injections, I am also convinced that the current approach may bias the results shown in figures 4-6. For instance, I found that the data presented in figures 7 and 8 (even without the NaCl session) are much more relevant to the overall (i.e., sickness changes the response profile of GC neurons) interpretation of the data.”*

**Response**: We appreciate R2’s point of view, here. Figures 4-6 contain analyses similar to those performed many times previously in the Katz lab (and in R2’s lab, we’d wager), and are central to the development of our understanding and to our ability to relate these data to the literature. But we performed the “held-unit” analyses (i.e., Figures 7 & 8) specifically because we realized that our interpretation of Figures 4-6 was rendered far more compelling when combined with a pair of held-unit interrogations. We agree that the manuscript would be weaker without those experiments. We have added text (pgs 12 & 16) to this effect, and added text throughout regarding the values and weaknesses of these different analyses. Furthermore, the details requested by R2 (including the fact that only 1 session of each type was recorded from each rat, thus reducing the value of moving the wires) are now included in the Methods section (pgs 5 & 6).

**R2**: *I wonder if the results observed in figures 4-6 are affected by potential differences in the sensory properties of different neurons across the GC (**the heterogeneity of GC responses within a recording session is highlighted by the authors in Fig 8; thus, we can expect even more heterogeneity across sessions and across animals).*

**Response**: While we admit that we are not 100% sure that we understand what R2 is driving at here, we agree that there is in GC (as there is everywhere in neuroscience) a good deal of response variability. We would argue, however, that this heterogeneity only adds to the power of our Figure 4-6 results, which are reliable despite this heterogeneity (and despite the fact that we did not “cherry pick” neurons that gave us particular responses)—the fact that the effects are strongly significant across the sample is a testament to their robustness in the face of variability. We have added a comment in the Results section making this argument (pg 12). Further, as can be seen in the updated Figure 1B, the consistency of our electrodes placement reduces the likelihood that our effects suffer overmuch from heterogeneity (see also Levitan, et al., 2019).

**R2:** *“I am somewhat confused by the criteria used to evaluate taste responsiveness. In the method session, the author indicated that "Data were collapsed all four tastes, such the ""responsivity"" indicates purely that a GC neurons responds to taste delivery and reveals nothing about taste specificity". If all the evoked responses to the different tastes are collapsed, what will happen if 1) a neuron shows an increase (or decrease) in response to only one taste and/or 2) a neuron is excited by two tastes and inhibited by the other two? What are the chances that these two hypothetical neurons will be deemed non-responsive based on your paired sample t-test (pre vs. post taste) after collapsing all the tastes?”*

**Response**: R2 is entirely correct to point out that “taste responsivity” and “taste specificity” have an uneasy relationship. There are several scenarios, including the two noted by R2, in which a neuron that fires in a taste-specific manner may fail to appear “taste responsive” when responses to all tastes are collapsed into one. Furthermore, a neuron that is deemed “taste responsive” can in certain scenarios fail to have significant responses to any individual tastes. It is the nature of statistical analysis, and is the reason that we included the statement that R2 quotes in the original manuscript.

We use both measures because both are used in the literature, but we are sympatico with the implication of R2’s line of questioning, which is: for all intents and purposes, the analysis of “taste-specificity” essentially supersedes any analysis of “taste responsiveness.” While we have left both in the revised manuscript (and have on pg 9 beefed up our statement concerning the relationship between the two analyses), we would be happy to drop the latter out, if that is R2’s wish.

**R2**: *“Figures 5 and 6 showed how sickness reduces taste discriminability (across all tastes; Fig. 5) and increases the palatability-relatedness (Fig. 6) of taste responses. I do not understand why the data in Figure 6 are not analyzed with the same analysis showed in Figure 5 (LDA). The author could have combined sweet-salt (good) and acid-bitter (bad) and run a time series of LDA to show that sickness allows the GC neurons to better discriminate good vs. bad taste, as per the main conclusion of the manuscript. Using different analyses to run "chemosensory" (Figure 5) and "palatability" (Figure 6) coding is confusing, to say the least.”*

**RESPONSE**: In deference to R2’s suggestion, we conducted an LDA to contrast palatable and aversive tastes. As expected, this analysis revealed no significant condition-specific differences, because GC ensembles do a great job (>62% accuracy post-early epoch) of distinguishing palatable tastes from aversive in both healthy and sick conditions. We haven’t included this in the revision, but we have tried in multiple places to better explain both the analyses that we used and the reasons for their use, explicitly noting that: 1) taste-specificity and palatability-relatedness are actually very different variables—the former makes use of any cell-specific differences in responses to different tastes, whereas the latter measures whether a particular pattern of responsiveness exists—a fact that makes it prudent to use very different analyses to reveal taste-specific and palatability-related firing (as we have done in many previous papers); and, perhaps more importantly 2) the impact of illness that we are observing and characterizing is actually a relatively subtle decrease in dimensionality—the ratio of the distances between “different-palatability” and “same-palatability” tastes becomes larger during illness—that can only be captured in analysis that goes beyond ‘simple’ assessment of palatability-relatedness.

**R2:** *“When evaluating how LiCl changed the "sign" of taste responses, the authors decided to ignore the first 300 ms of post taste activity. They justified this choice by indicating that evoked responses within 300 ms after stimulus delivery do not contain chemosensory information. This is absolutely true in the case of the many reports from the authors (and others) when tastes are delivered intra-orally. However it is not the case when stimuli are licked from a spout (**Stapleton 2006, 2007; Gutierrez 2010; Graham 2014; Bouaichi 2020; Dikecligil 2020). I suggest that the author highlight that 300ms of post-taste activity is seldom chemosensory in the context of intra-oral cannulae delivery. Also, this seems like a lost opportunity. If there is no chemosensory information in that time window, the author should use it as a control and show that there is no change in the sign of the responses in that early epoch in LiCl.”*

**Response**: Both of R2’s suggestions are wise. In the resubmission, we repeatedly note (pgs 8 ,9, & 14) this difference between ours and “active tasting” paradigms (we apologize for having failed to mention these articles in the original submission); in addition, we now include an analysis (pg 13) showing that the first 200 msec of our GC responses are devoid of taste content (see our response to R1) and thereby “unflippable” of content.

**R2:** *“One of the main results of this paper is that, sickness causes the firing properties of GC neurons to collapse to a pure good vs. bad code of whether or not a taste is palatable. Would this be behaviorally relevant? Is the cortex (or the entire forebrain for that matter, [see for example Grill and Norgren 1978]) needed to compute such basic and salient sensory information? Could the authors comment on these points in the Discussion section?”*

**Response**: We are happy to do so. The Discussion section (pg 16) now extensively discusses the evidence that, while basic palatability behaviors can be driven by a purely brainstem circuit (Grill and Norgren, 1978), GC activity plays an active role in driving such behaviors *in situ* (e.g., Sadacca, et al., 2016; Mukherjee, et al., 2019). As part of this paragraph, we note the many other sensorimotor systems (refs from bottom of pg 15 in Mukherjee) in which simple CPGs are similarly capable of functioning in isolation but subject to top-down modulation *in situ*.

**R2:** *“There is almost no information on how the single neurons are sorted. Is the SNR the only quality metric used? I think it would be better to add some more details even if they are described elsewhere. Likewise, I would replace all the "data not shown" with the actual data.”*

**Response**: We have, as requested, greatly expanded the description of our spike sorting routine (pg 7), and have removed references to unshown data.

**R2:** “It would be helpful to see some examples of single neuron activity (rasters and PSTHs) associated with either Figure 3 or 4.”

**Response**: As noted above, we have added representative single-neuron data to Figures 4 & 8.

**R2:** *“The author provide a relatively low-quality image of one "representative" electrode track (please, do add a scale bar). It might be beneficial to show all the electrodes tracks to give the reader a better sense of the recording locations.”*

**Response**: Another very good suggestion; thank you, R2. We have swapped in a higher-resolution image, and added a schematic showing all of the electrode placements, complete with scale bar (Figure 1).

**R2:** *“It is not clear how many single units were recorded per animal and per session (NaCl and LiCl). The authors should provide this information.”*

**Response**: As noted above in our response to R1, we have made multiple changes to the revision to correct this oversight.

**R2:** *“The description of Fig. 2B in the text is wrong.”*

**Response**: Whoops. We’re embarrassed. We’ve fixed the problem.

**Reviewer 3 (R3)**

**R3:** *“The authors refer to taste cortex; this is not a clear term. I suggest they will talk about the relevant area in the insular cortex: Left? Right? anterior? Agranualr? disgranular? granular?, inner or outer layers. We know much more about subdivisions within the IC that should be take into account. “*

**Response**: We now define the location of gustatory cortex more completely in the Introduction (pg 4), and again in the Methods section (pg 5).

**R3:** *“What does it mean that half cases amplitude of activity was reduced and half was increased?“*

**Response**: We expect that R3 is referring to the section of the Results describing the LFP data. To ensure that we’ve answered the question thoroughly, we’ve altered the Results section to be clearer about what we actually observed (pg 10).

**R3:** *“Can you call the measured activity spontaneous?“*

**Response**: We appreciate what we suspect is the question behind R3’s question; neural activity is almost never truly spontaneous … and certainly not when one is evaluating what happens after injecting LiCl. For simplicity’s sake, but have expunged the manuscript of the word “spontaneous.” This decision has led us to update the title, as well.

**R3:** *“Does the behavioral facial expression the authors recorded can be analyzed as in Gogola to better define sickness state?“*

**Response**: Our video data are inadequate for these purposes, alas. It is also unclear whether this technique would work in this circumstance, in which we induced only mild illness, although it would be absolutely worthwhile to find out!

**R3:** *“The fact that data for fig 2 and 3 was collected from different groups is less convincing. The difference in time for changes in power or behavior within groups could use to validate the results. One would assume that first will appear the change in power and only later in behavior. Data from each rat could validate this point (i.e. the connection between change in behavior and change in power).“*

**Response**: We appreciate R3’s concern, and wish that we could resolve small timing differences in brain and behavior. The data clearly show that the changes in brain and behavior emerge in tandem, however—a conclusion that we would argue is actually more conservative and compelling when reached on the basis of datasets that were collected separately. The fact that the onset of illness-related behaviors and spectral changes of cortical activity were approximately simultaneous even in animals tested at different times, despite the attendant variability of conditions, strongly suggests that the two are related.

To bolster this argument with data, and in appreciation of R3’s position, we have for the revision performed a Bayesian analysis (detailed in Methods, pg 8) of the likelihood of such close synchronization of processes by chance (pg 11); this analysis confirms the surprisingly coupled nature of the changes, and thereby the fact that they are related.

**R3:** *“Fig 4, though the authors claim only the middle epoch was sig different between saline and LiCl, all epochs show same direction and about the same size? Can the authors claim for specificity of the middle epoch? … Following the above, why the authors did not analyze the time scale between 1 and 1.5 second? I would assume they find also a sig difference in fraction of trials correct at the "post" late epoch. What does it mean? “*

**Response**: We find ourselves agreeing whole-heartedly with R3’s observations, and we have therefore overhauled Figure 4, adding representative data and removing much of the questionable analysis and presentation (which had added little to the story).

**R3:** “Fig 1 and 2 can unify.”

**Response**: We would rather not do this, because: 1) both Figures are already multi-panel (in fact, Figure 1 has grown in size—see below), and 2) we prefer that each of our figures be devoted to a single concept, and Figures 1 & 2 have different main points. Having said this, we would be happy to combine the two if R3 and the editor agree that it is important that we do so!

**R3:** *“The author should present all brain imaging following all treatments.”*

**Response**: R2 made a related request, with which we have gratefully complied: Figure 1 now includes the requested information.

**Reviewer 4 (R4)**

**R4:** *“As mentioned above, I was surprised how the authors did not relate their findings to learning about CTAs. A recent paper by the Moran group looked at changes in GC processing following CTA. It seems appropriate to discuss their findings given what the authors found here. Specifically, these results indicate that several of the findings by the Moran group are not necessarily due to changes in learning, but are changes in activity induced by the illness that set the stage for learning. It seems that a re-assessment of previous results on changes in activity within GC during CTA, both from the Katz group as well the recent study from the Moran group, would be very worthwhile in light of the findings in this paper. Of course, a full consideration might require a separate review, but at minimum a mention seems pertinent to the discussion. “*

**RESPONSE**: We thank the reviewer for this suggestion. We had failed to reference this paper from the Moran group, which was published after we submitted our work for review. However, upon reading it, we agree that our paper and their’s together provide a rich accounting of the GC changes that follow LiCl injection. We actually think that the two papers dovetail nicely, with Moran’s work suggesting how LiCl-induced GC changes might promote the acquisition of CTA. We have updated the Discussion (pg 15) to incorporate our thoughts on this work.

**R4**: *“The analyses performed in reference to figure 4 are a bit confusing and may lead the reader down the wrong interpretation of the study. My general understanding was that the authors found no general changes in activity between conditions (the specifics of this analysis were a bit unclear, see below), although they did find changes in the percentages of neurons that were significantly inhibited/excited/unchanged. The authors then reanalyze changes in activity across these groups and find modest activity changes. As a reader, it seemed that the authors were stretching to show a significant effect on activity across the two conditions, yet this seemed to be a moot point after reading the rest of the paper as there were clear and robust changes in the coding of taste stimuli across conditions. It's not clear that figure 4 as a whole adds substantially to the paper and even perhaps acts as something of a red herring. The small changes in firing rates between the 'excited' groups is underwhelming, particularly when there are more neurons in the group with the smaller average. It seems some other analysis would fit better here. Maybe a comparison of the empirical cumulative distributions of the firing rates between conditions? As for confusion regarding the analysis performed: What exactly were the authors comparing here? Is it baseline vs evoked activity for each neuron for each taste?: 'As an initial look at how illness impacts taste-evoked spiking activity of GC neurons, we evaluated the quantity and magnitudes of responses elicited by tastes delivered directly into the mouth via IOC. Paired-sampled t-tests (with Bonferroni correction for multiple comparisons) revealed that LiCl did not significantly impact the magnitude of responses in a taste-specific manner (ps <0.05).' Wording here is confusing. By putting the (within condition) it sounds like the averages are for each condition. Is it that you took the average differences of activity, compared to baseline, for all tastes for each neuron and performed a Chi square test?: "For this reason, we averaged taste responses (within condition) and a subsequent chi-square test of independence revealed a significant relationship between condition and direction of response on neuron count“*

**Response**: We find ourselves in general agreement with R4’s insights. As noted above in our responses to the other reviewers, we have added the requested information to the Methods section (pg 6), and have overhauled Figure 4 so that it does a better job of communicating the important point without introducing questionable and distracting information.

**R4:** *“I'm not sure I understood the following argument. Could the authors provide a bit more clarification?: 'While one could speculate that illness' effect on GC μ power merely represents changes in firing rates (FRs) across time, a two-way ANOVA revealed a there was no significant interaction between the experimental group (saline-LiCl versus saline- saline) and quartile on change in FR (F(3, 33) = 2.39, p = 0.09); this suggests that our observed effect on μ is not simply a gating of firing rate direction, but rather a matter of synchrony being modulated.“*

**Response**: We have simplified this inarguably confusing sentence (pg 11).

**R4:** *“This may relate to the above. The authors make a clear point that the changes in mu power are transient, yet the observed effects in the paper raise the question about whether a good portion of the observations may be due to changes of evoked taste activity that occurs within mu. The reason I'm suggesting this is that prior work from the Katz group has shown that tastes delivered during mu results in a similar effect which is observed in this paper - that discrimination across similarly palatable tastes decreases while palatability coding increases. This may not necessarily matter to the main point of the paper, but if more tastes are delivered during the mu state, this result would certainly fit with prior work from the lab.“*

**Response**: We thank R4 for this suggestion, which we are embarrassed to have not thought of on our own. The revised Discussion section now contains text devoted to a comparison between the current results and those from Fontanini & Katz, 2006 (pg 16).

**R4:** *“In Figure 1A, did the authors forget to include the control group, shouldn't there also be a group of rats with saline injections at the same time as the LiCl injections? If I'm not misunderstanding the design, I would certainly add this since readers might assume that all comparisons were to the NaCl injections on the second day. “*

**Response**: We thank R4 for pointing this out; we have updated Figure 1A to more accurately reflect our design.

**R4:** *“Figure 2B references in the results section are flipped (top - control; bottom - experimental)“*

**Response**: Once again we are indebted to R4 for pointing something out that we should have caught ourselves. We have updated the manuscript to fix Figure 2B.

**R4:** *“In the results, when discussing figure 2C, the authors mention 'subsequent testing'. What exactly was performed here? “*

**Response**: We have modified this sentence in the Results section (pg 11) to be about “subsequent analysis”—specifically a one-way ANOVA to test the differences within bins.

**R4:** *“The authors ran an LDA classifier and compared the results across conditions using a Mann-Whitney U test (page 9). My guess is that a classifier was run for each neuron rather than on a pseudo-ensemble, but it's not clear which is the case either looking in the results or the methods sections. If the latter, please add more clarification about how separate samples, and how many, were included in the Mann-Whitney U-Test “*

**Response**: We apologize for the lack of clarity. We have updated the Methods section (pg 9) to specify that the LDA test was run on each simultaneously-recorded ensemble (i.e., the n was the number of sessions), and the Mann-Whitney U test was then used (because the data were deemed inappropriate for parametric statistics) to compare the average result in illness sessions to that in healthy sessions.

**R4:** *“Editing error: 'a two-way ANOVA revealed a there was no“*

**Response**: Thank you for this catch. We have fixed the mistake.
