# Supplementary material for: LiCl-induced sickness modulates rat gustatory cortical responses
Source: PLoS Biol. 2022 Jul 25;20(7):e3001537. doi: 10.1371/journal.pbio.3001537 (PMC9352195; doi:10.1371/journal.pbio.3001537)
Supplement: Attachment — Submitted filename: Stone et al - Response letter 2.docx [file pbio.3001537.s009.docx]

We are grateful to the editors for inviting this 2^nd^ revision, and to the reviewers for their support and continued willingness to help us improve the manuscript. It seems that there are two real concerns remaining, only one of which—a criticism offered by R1 alone—requires an extensive response. Below, we address this concern at length, our goal being to provide maximal clarity with regard to our appreciation of the concern, the range of possible responses to the concern, the problems with each possible response, and our ultimate action. The 2^nd^, minor concern is handled after.

R1’s primary concern is essentially the possibility that LiCl could independently cause illness behaviors (that we have measured in one group of rats) and cortical changes (that we have measured in a different group of rats)—a hypothesis that, while unlikely, is entirely deserving of consideration. R1 suggests that our argument that cortical changes are in fact related to illness (rather than being caused by direct action of LiCl) would be more compelling if we were to collect those data from the same rats. Below, we explain at length why we disagree, but we then follow this explanation with changes that we have nonetheless made to the manuscript in appreciation of R1’s stance.

Alas, the experiment that R1 recommends would not actually yield valid, interpretable results, for the simple reason that the animal’s head has to be hooked to an electrode and cannula harness for purposes of electrophysiological data collection. Remember that we have deliberately caused very mild illness, to ensure that illness-related behaviors would be subtle enough to not cause cortical changes themselves (changes that would obscure any changes directly caused by the illness). This subtle behavioral sign (altered rearing behavior), observable in untethered rats, does not even occur in animals attached to this relatively awkward electrode/cannula harness. “*Ephys” rats don’t show rearing behavior, not because they aren’t ill, but because the harness necessary for electrophysiology weighs them down enough that it inhibits this behavior*; **a negative result in this experiment would therefore be uninterpretable**.

But what if we WERE to see illness-related behavior in these animals, despite the harness? Would seeing both behavioral and neural changes in the same animals disprove R1’s concern that LiCl might cause the two types of changes independently? The answer is no. *Even if we were to see both altered rearing behavior and altered cortical responses in the same LiCl-treated rats, it would still be just as possible that the LiCl was causing the two changes independently*; **that is, neither a negative nor a positive result in this experiment would settle anything** – R1’s proposed experiment simply would not allow us to more conclusively rule out one interpretation or the other.

So what would we need to do to definitively disprove the alternative hypothesis? One solution might be to perform an additional set of experiments in which we somehow block LiCl’s illness-causing effect, and test whether this manipulation also blocks cortical changes. This would be a major and highly difficult project – a huge expenditure of time and money done to rule out a highly unlikely interpretation of our full set of results (see below). Furthermore, because the alternative hypothesis is only 100% ruled out if LiCl NEVER causes cortical changes unless it also causes illness, we’d need to keep going until we’d tested every possible pathway. **Testing whether LiCl is even *capable* of causing cortical changes independently of illness would be an entire paper on its own**.

In light of the above, we would argue that the best solution is actually to do what we have done. Specifically, we have tested a rigorous and risky prediction: rather than just predicting that LiCl would both make the rats ill and change cortical activity, *we specifically predicted that LiCl would do both at about the same latencies*. **Given the success of this prediction (see Figures 2, 3, & 7), the most likely explanation (by far) is that the two are linked – that LiCl-induced illness is reflected in cortical activity**. This conclusion holds regardless of whether the behavioral and neural datasets were collected from the same rats.

Having made the above argument, we nonetheless cannot fail to appreciate R1’s stance on this issue – the fact that we can never be 100% sure that the impact of LiCl on cortical coding specifically reflects the fact that LiCl makes the rat ill. We have therefore: 1) added a paragraph to the Discussion regarding the appropriate level of assurance (high, but not 100%) that can be reached on the basis of our results; 2) added text to the Results that foreshadows this Discussion; 3) changed statements throughout to modestly suggest that GC changes are LiCl- (rather than “illness-“) induced; and 4) for a 2^nd^ time adjusted the title of the manuscript, which is now “Systemic administration of illness-inducing LiCl modulates field potential spectral power and response dynamics in rat gustatory cortex.”

The one other issue to be resolved is the reasonable concern expressed by both R1 and R2 regarding the representativeness of the PSTHs shown in Figure 4. We apologize for the confusion that these panels created and are grateful to the reviewers for their eagle eyes. We have modified the format of the figure and revised the figure legend to enhance the clarification that the GC neurons in each PSTH panel are distinct, but not pairs of, neurons which unsurprisingly have varying baseline firing rates. We have also updated the panels with neurons that illustrate more typical responses, that is, a lack of taste-driven differences in firing rates between 0-200ms (we admit to having gotten so excited about the representativeness of their later-epoch responses that we missed the problem). We refer the editors and reviewers to Figures 5 and 6, which confirm that little to no taste-specificity is apparent in the earliest responses overall.
