# Supplementary material for: LiCl-induced sickness modulates rat gustatory cortical responses
Source: PLoS Biol. 2022 Jul 25;20(7):e3001537. doi: 10.1371/journal.pbio.3001537 (PMC9352195; doi:10.1371/journal.pbio.3001537)
Supplement: Peer review history

# Editor report: Decision Letter 0

**DOI:** 10.1371/journal.pbio.3001537.r001

**Authors:** Gabriel Gasque

10 Jan 2022

Dear Dr Katz, 

Thank you for submitting your manuscript entitled "LiCl-induced sickness modulates spontaneous activity and response dynamics in rat gustatory cortex." for consideration as a Research Article by PLOS Biology.

Your manuscript has now been evaluated by the PLOS Biology editorial staff, as well as by an academic editor with relevant expertise, and I am writing to let you know that we would like to send your submission out for external peer review. Please accept my apologies for the delay in communicating this decision to you. We are working through our post-holiday backlog. 

Before we can send your manuscript to reviewers, we need you to complete your submission by providing the metadata that is required for full assessment. To this end, please login to Editorial Manager where you will find the paper in the 'Submissions Needing Revisions' folder on your homepage. Please click 'Revise Submission' from the Action Links and complete all additional questions in the submission questionnaire.

Once your full submission is complete, your paper will undergo a series of checks in preparation for peer review. Once your manuscript has passed the checks it will be sent out for review. To provide the metadata for your submission, please Login to Editorial Manager (https://www.editorialmanager.com/pbiology) within two working days, i.e. by Jan 12 2022 11:59PM.

If your manuscript has been previously reviewed at another journal, PLOS Biology is willing to work with those reviews in order to avoid re-starting the process. Submission of the previous reviews is entirely optional and our ability to use them effectively will depend on the willingness of the previous journal to confirm the content of the reports and share the reviewer identities. Please note that we reserve the right to invite additional reviewers if we consider that additional/independent reviewers are needed, although we aim to avoid this as far as possible. In our experience, working with previous reviews does save time. 

If you would like to send previous reviewer reports to us, please email me at ggasque@plos.org to let me know, including the name of the previous journal and the manuscript ID the study was given, as well as attaching a point-by-point response to reviewers that details how you have or plan to address the reviewers' concerns. 

During the process of completing your manuscript submission, you will be invited to opt-in to posting your pre-review manuscript as a bioRxiv preprint. Visit http://journals.plos.org/plosbiology/s/preprints for full details. If you consent to posting your current manuscript as a preprint, please upload a single Preprint PDF. 

Given the disruptions resulting from the ongoing COVID-19 pandemic, please expect some delays in the editorial process. We apologise in advance for any inconvenience caused and will do our best to minimize impact as far as possible.

Feel free to email us at plosbiology@plos.org if you have any queries relating to your submission.

Kind regards,

Gabriel

Gabriel Gasque

Senior Editor

PLOS Biology

ggasque@plos.org

# Peer review: Decision Letter 1

**DOI:** 10.1371/journal.pbio.3001537.r002

**Authors:** Gabriel Gasque

7 Feb 2022

Dear Dr Katz,

Thank you for submitting your manuscript "LiCl-induced sickness modulates spontaneous activity and response dynamics in rat gustatory cortex." for consideration as a Research Article at PLOS Biology. Your manuscript has been evaluated by the PLOS Biology editors, by an Academic Editor with relevant expertise, and by four independent reviewers.

In light of the reviews (below), we will not be able to accept the current version of the manuscript, but we would welcome re-submission of a much-revised version that takes into account the reviewers' comments. We cannot make any decision about publication until we have seen the revised manuscript and your response to the reviewers' comments. Your revised manuscript is also likely to be sent for further evaluation by the reviewers.

We expect to receive your revised manuscript within 3 months. 

Please email us (plosbiology@plos.org) if you have any questions or concerns, or would like to request an extension. At this stage, your manuscript remains formally under active consideration at our journal; please notify us by email if you do not intend to submit a revision so that we may end consideration of the manuscript at PLOS Biology.

**IMPORTANT - SUBMITTING YOUR REVISION**

Your revisions should address the specific points made by each reviewer. As you will see, the reviewers agree that your study is potentially important but point out a few issues that should be addressed. Most importantly, there is a general lack of clarity in the description of the results and methods. The referees also make a number of suggestions to further improve the manuscript and its conclusions. There is considerable overlap between reviews, and you should pay special attention to all major comments raised by reviewer 1, as well as points 3 and 4 by reviewer 2 (point 4 is related to reviewer 1's point 3).

Please submit the following files along with your revised manuscript:

1. A 'Response to Reviewers' file - this should detail your responses to the editorial requests, present a point-by-point response to all of the reviewers' comments, and indicate the changes made to the manuscript. 

*NOTE: In your point by point response to the reviewers, please provide the full context of each review. Do not selectively quote paragraphs or sentences to reply to. The entire set of reviewer comments should be present in full and each specific point should be responded to individually, point by point.

You should also cite any additional relevant literature that has been published since the original submission and mention any additional citations in your response. 

2. In addition to a clean copy of the manuscript, please also upload a 'track-changes' version of your manuscript that specifies the edits made. This should be uploaded as a "Related" file type. 

*Re-submission Checklist*

When you are ready to resubmit your revised manuscript, please refer to this re-submission checklist: https://plos.io/Biology_Checklist

To submit a revised version of your manuscript, please go to https://www.editorialmanager.com/pbiology/ and log in as an Author. Click the link labelled 'Submissions Needing Revision' where you will find your submission record. 

Please make sure to read the following important policies and guidelines while preparing your revision:

*Published Peer Review*

Please note while forming your response, if your article is accepted, you may have the opportunity to make the peer review history publicly available. The record will include editor decision letters (with reviews) and your responses to reviewer comments. If eligible, we will contact you to opt in or out. Please see here for more details:

https://blogs.plos.org/plos/2019/05/plos-journals-now-open-for-published-peer-review/

*PLOS Data Policy*

Please note that as a condition of publication PLOS' data policy (http://journals.plos.org/plosbiology/s/data-availability) requires that you make available all data used to draw the conclusions arrived at in your manuscript. If you have not already done so, you must include any data used in your manuscript either in appropriate repositories, within the body of the manuscript, or as supporting information (N.B. this includes any numerical values that were used to generate graphs, histograms etc.). For an example see here: http://www.plosbiology.org/article/info%3Adoi%2F10.1371%2Fjournal.pbio.1001908#s5

*Blot and Gel Data Policy*

We require the original, uncropped and minimally adjusted images supporting all blot and gel results reported in an article's figures or Supporting Information files. We will require these files before a manuscript can be accepted so please prepare them now, if you have not already uploaded them. Please carefully read our guidelines for how to prepare and upload this data: https://journals.plos.org/plosbiology/s/figures#loc-blot-and-gel-reporting-requirements

*Protocols deposition*

To enhance the reproducibility of your results, we recommend that if applicable you deposit your laboratory protocols in protocols.io, where a protocol can be assigned its own identifier (DOI) such that it can be cited independently in the future. Additionally, PLOS ONE offers an option for publishing peer-reviewed Lab Protocol articles, which describe protocols hosted on protocols.io. Read more information on sharing protocols at https://plos.org/protocols?utm_medium=editorial-email&utm_source=authorletters&utm_campaign=protocols

Thank you again for your submission to our journal. We hope that our editorial process has been constructive thus far, and we welcome your feedback at any time. Please don't hesitate to contact us if you have any questions or comments.

Sincerely,

Gabriel

Gabriel Gasque

Senior Editor

PLOS Biology

ggasque@plos.org

*****************************************************

REVIEWS:

Reviewer #1: Comments:

LiCl-induced conditioned taste aversion is a fundamental mechanism to study taste-visceral interactions. However, how malaise impacts cortical processing of this sensory information is unknown. This report provides novel insights into the effects of LiCl-induced malaise on neural activity in the gustatory cortex. The authors show that spontaneous changes in LFPs at the µ frequency (7-12 Hz) arise on a similar timescale as the onset of illness-related behaviors. Furthermore, LiCl-malaise altered single-unit responses in the gustatory cortex; with reductions in response magnitude and discriminability, but an enhancement in the representation of taste palatability. In summary, the authors detail how a change in state (malaise) that reduces consumption alters the representation of taste signals in sensory cortex.

While the question is important to the field and the authors interpretations are measured and interesting, I had a hard time grasping exactly what datasets were being analyzed throughout the Results. This made judging the impact of the findings extremely difficult. However, the authors can alleviate my concerns by addressing the following points. 

Major Concerns:

1. Both in the text of the Results and the figures, there is a dearth of experimental detail related to the number of animals and/or neurons used in the analyses. As far as I could tell, only the section titled "Illness-related changes in taste coding occur in single neurons", and its corresponding figure (Fig. 7), lists the number of animals (n=3), number of total recorded neurons (n=74), and the subset of taste responsive neurons (n=56) used for the analyses. It is entirely unclear to me what populations of single-units are being compared in the section titled "Illness causes taste responses to resemble a good-bad distinction" (Fig. 4, Fig. 5, and Fig. 6). Are these populations from the same rats, but different days? If so, are they recorded at the same depth? Are the analyses performed across all neurons, only taste responsive neurons, or only the taste responsive neurons with stable waveforms across days (putative single-neurons)? What constitutes a "change in firing rate"? Is this a neuron by neuron comparison in taste-evoked activity between LiCl- and Saline-sessions? Or is this a comparison between all recorded neurons from the different sessions? Is baseline firing rate taken into account, because a 5Hz change in activity may impact the system more for a neuron with a 5hz baseline than one with a 50Hz baseline. The lack of specific information impedes a clear understanding of the results and mars the author's interpretations

2. Relatedly, the presentation of the data would be significantly enhanced by the addition of representative examples to the figures describing the single-unit activity (i.e. Fig. 4, Fig. 6) and especially the figures showing changes in single-unit activity within a session (Fig. 7 and Fig. 8).

3. Would the authors please explain why the first 300ms of the taste evoked activity is removed for some analysis (i.e. directionality), but then used as a key component of the analyses of taste epochs. The timeframe of 300ms seems questionable, given the many studies from this group showing that taste identity begins to emerge ~200ms after stimulus delivery (Katz et al., 2001; Fontanini and Katz, 2006; Sadacca et al., 2012; Sadacca et al., 2016). What is the justification for ignoring data for one analyses, but including it for others? Does including the data eliminate the significant difference in the directionality analyses?

Minor concerns:

1. I agree that it's interesting the illness-related behavior from a separate group of rats correlates with the onset of changes in LFP. However, can the authors provide any evidence that the rats in the electrophysiological group experienced malaise? Especially since only 5 of the 7 exhibited a significant change in LFP? For example, LiCl-induced malaise can evoke a number of aversive oromotor and somatic responses (Spector et al., 1988), including gape, chin rub, head shake, and forelimb flail. Did either group (ephys. or behavior) perform more of these behaviors after LiCl-induced malise compared to Saline controls?

2. For the analyses represented by Figure 8, the authors say "pre-stimulus firing rates fall generally along the diagonal, demonstrating that spontaneous firing rates do not differ strongly between condition". Individual variability aside, do the spontaneous firing rates of putative single neurons (as a population) differ between the LiCl- and Saline-sessions? Statistical confirmation would further support the stability of the population across sessions. 

3. The authors should take care when describing activity suppressed by taste delivery as "inhibitory". I have received this same criticism in the past because some readers wrongly assume that the authors are suggesting that these neurons are themselves inhibitory. 

4. The manuscript would benefit from a spelling and grammar check, as there were a number of typos throughout the article. 

Reviewer #2: In this paper, Stone et al., made electrophysiological recordings from the gustatory cortex (GC) of healthy

and ill (due to LiCl injections) female rats. They evaluated how a behavioral state (illness) alters the way GC

neurons represent the chemosensory and hedonic properties of tastants delivered via intra-oral cannulae. Overall,

the results show that being in a sickness state changes basic properties of the sensory responses to basic taste

qualities. Many others reports (also from the author's laboratory) have already showed that GC responses are

plastic and are strongly affected by behavioral states (even behavioral states less "salient" than illness behavior).

Nevertheless, this is a fine paper that describes how GC neurons alter their taste-evoked spiking activity after

LiCl injection - changes that somehow correlate with the onset of sickness behavior. The authors might consider

the following points:

1 - It would be helpful to know some more information about the recording strategy to better interpret the data

presented in figures 4-6. That is, were the electrodes lowered after each NaCl and LiCl recording sessions to

record new ensembles of GC neurons? If that is the case, I have two main concerns: 1) I wonder if the results

observed in figures 4-6 are affected by potential differences in the sensory properties of different neurons across

the GC (the heterogeneity of GC responses within a recording session is highlighted by the authors in Fig 8;

thus, we can expect even more heterogeneity across sessions and across animals). 2) While I do understand the

challenges of an experimental design in which the same neurons are recorded (and held) during and after NaCl

and LiCl injections, I am also convinced that the current approach may bias the results shown in figures 4-6.

For instance, I found that the data presented in figures 7 and 8 (even without the NaCl session) are much more

relevant to the overall (i.e., sickness changes the response profile of GC neurons) interpretation of the data.

2 - I am somewhat confused by the criteria used to evaluate taste responsiveness. In the method session, the

author indicated that "Data were collapsed all four tastes, such the ""responsivity"" indicates purely that a GC

neurons responds to taste delivery and reveals nothing about taste specificity". If all the evoked responses to the

different tastes are collapsed, what will happen if 1) a neuron shows an increase (or decrease) in response to only

one taste and/or 2) a neuron is excited by two tastes and inhibited by the other two? What are the chances that

these two hypothetical neurons will be deemed non-responsive based on your paired sample t-test (pre vs. post

taste) after collapsing all the tastes?

3 - Figures 5 and 6 showed how sickness reduces taste discriminability (across all tastes; Fig. 5) and increases

the palatability-relatedness (Fig. 6) of taste responses. I do not understand why the data in Figure 6 are not analyzed

with the same analysis showed in Figure 5 (LDA). The author could have combined sweet-salt (good) and

acid-bitter (bad) and run a time series of LDA to show that sickness allows the GC neurons to better discriminate

good vs. bad taste, as per the main conclusion of the manuscript. Using different analyses to run "chemosensory"

(Figure 5) and "palatability" (Figure 6) coding is confusing, to say the least.

4 - When evaluating how LiCl changed the "sign" of taste responses, the authors decided to ignore the first 300 ms

of post taste activity. They justified this choice by indicating that evoked responses within 300 ms after stimulus

delivery do not contain chemosensory information. This is absolutely true in the case of the many reports from

the authors (and others) when tastes are delivered intra-orally. However it is not the case when stimuli are licked

from a spout (Stapleton 2006, 2007; Gutierrez 2010; Graham 2014; Bouaichi 2020; Dikecligil 2020). I suggest

that the author highlight that 300ms of post-taste activity is seldom chemosensory in the context of intra-oral

cannulae delivery. Also, this seems like a lost opportunity. If there is no chemosensory information in that time

window, the author should use it as a control and show that there is no change in the sign of the responses in that

early epoch in LiCl.

5 - One of the main results of this paper is that, sickness causes the firing properties of GC neurons to collapse

to a pure good vs. bad code of whether or not a taste is palatable. Would this be behaviorally relevant? Is the

cortex (or the entire forebrain for that matter, [see for example Grill and Norgren 1978]) needed to compute such

basic and salient sensory information? Could the authors comment on these points in the Discussion section?

6 - There is almost no information on how the single neurons are sorted. Is the SNR the only quality metric

used? I think it would be better to add some more details even if they are described elsewhere. Likewise, I would

replace all the "data not shown" with the actual data.

7 - It would be helpful to see some examples of single neuron activity (rasters and PSTHs) associated with

either Figure 3 or 4.

8 - The author provide a relatively low-quality image of one "representative" electrode track (please, do add a

scale bar). It might be beneficial to show all the electrodes tracks to give the reader a better sense of the recording

locations.

9 - It is not clear how many single units were recorded per animal and per session (NaCl and LiCl). The

authors should provide this information.

Minor:

1 - The description of Fig. 2B in the text is wrong.

2 - Overall, the manuscript is a pleasure to read because it is very well written. There are a few places where I

can find typos but these are super-minor (e.g., decide to use "μ" or "mu" and be consistent).

Reviewer #3: Stone et al., recorded single unit activity and LFP from IC of rats in correlation with CTA formation (i.e. before and during drinking novel taste and before and after LiCl injection i.p. as UCS). They report alteration in LFP power 11 min following the injection together with reduction in single neuron taste response. In addition, they report for reduced discriminability and enhancement in palatability. This is interesting novel and an important work. The research is based heavily on previous publication from the same lab. The authors recorded from small numbers of rats but it seems that the rigor analysis they did justify such a small number. A major point the authors do not take into account in all figures and analysis that it is not only the presentation of the taste and its valance! One would assume that a learning process of an association between malaise and taste takes place also within the very same neurons they record from. Its not only about taste but about prediction and association. This make the interpretations for the data more complex (I propose they will refer to this in the discussion). Nevertheless, the manuscript is important and innovative. 

Major

1. The authors refer to taste cortex; this is not a clear term. I suggest they will talk about the relevant area in the insular cortex: Left? Right? anterior? Agranualr? disgranular? granular?, inner or outer layers. We know much more about subdivisions within the IC that should be take into account. 

2. What does it mean that half cases amplitude of activity was reduced and half was increased? 

3. Can you call the measured activity spontaneous? 

4. Does the behavioral facial expression the authors recorded can be analyzed as in Gogola to better define sickness state? 

5. The fact that data for fig 2 and 3 was collected from different groups is less convincing. The difference in time for changes in power or behavior within groups could use to validate the results. One would assume that first will appear the change in power and only later in behavior. Data from each rat could validate this point (i.e. the connection between change in behavior and change in power). 

6. Fig 4, though the authors claim only the middle epoch was sig different between saline and LiCl, all epochs show same direction and about the same size? Can the authors claim for specificity of the middle epoch? 

7. Following the above in Fig 4, why the authors did not analyze the time scale between 1 and 1.5 second? I would assume they find also a sig difference in fraction of trials correct at the "post" late epoch. What does it mean?

Minor

1. Fig 1 and 2 can unify.

2. The author should present all brain imaging following all treatments. 

Reviewer #4: In this manuscript, Stone et al. assess the impacts of LiCl induced illness on neural activity in the gustatory cortex (GC). They found that LiCl injections resulted in a robust change in the mu frequency range, ~15 min post injection, within the local field potentials (LFP), which corresponded to a change in the activity of the rats as assessed by the presence of rearing behavior. The authors then analyzed taste-evoked activity within GC following illness and found subtle, yet meaningful, effects on GC activity. Notable changes included a reduction in the number of significantly inhibited neurons, a reduction in taste discriminability within GC neurons, and an increase in taste palatability. The overall decrease in discriminability and increase in palatability appear to be very robust. These findings, along with the distinction of a specific cluster of neurons within GC that seem to be most strongly affected by the illness, are very intriguing and cast substantial light onto the general process of illness-induced activity changes in the brain.

This work provides an excellent backdrop for understanding the baseline effects of how a gastro-induced illness may influence activity within GC. What I find to be the most intriguing and insightful aspect of the design of this experiment, which the authors conspicuously did not raise in the introduction or discussion, is how it provides a fundamental starting point for studying learning of taste-illness pairings, or conditioned taste aversion. This experiment reveals how illness itself affects coding in GC without the taste-illness learning process interacting with the effects. More specifically, the changes in palatability coding in the absence of taste-illness learning is extremely intriguing and helps constrain the possible mechanisms that may lead to this type of learning. I'll leave it to the authors whether they feel it is worth discussing this in the paper. 

In general the manuscript was well-written and analysis were on-point and well-performed, although there were a few parts in the manuscript where it was a bit unclear on the specifics of the design and the analyses performed. These are detailed below. Otherwise, my concerns revolve more around the discussion and interpretation of the results.

1. As mentioned above, I was surprised how the authors did not relate their findings to learning about CTAs. A recent paper by the Moran group looked at changes in GC processing following CTA. It seems appropriate to discuss their findings given what the authors found here. Specifically, these results indicate that several of the findings by the Moran group are not necessarily due to changes in learning, but are changes in activity induced by the illness that set the stage for learning. It seems that a re-assessment of previous results on changes in activity within GC during CTA, both from the Katz group as well the recent study from the Moran group, would be very worthwhile in light of the findings in this paper. Of course, a full consideration might require a separate review, but at minimum a mention seems pertinent to the discussion.

2. The analyses performed in reference to figure 4 are a bit confusing and may lead the reader down the wrong interpretation of the study. My general understanding was that the authors found no general changes in activity between conditions (the specifics of this analysis were a bit unclear, see below), although they did find changes in the percentages of neurons that were significantly inhibited/excited/unchanged. The authors then reanalyze changes in activity across these groups and find modest activity changes. As a reader, it seemed that the authors were stretching to show a significant effect on activity across the two conditions, yet this seemed to be a moot point after reading the rest of the paper as there were clear and robust changes in the coding of taste stimuli across conditions. It's not clear that figure 4 as a whole adds substantially to the paper and even perhaps acts as something of a red herring. The small changes in firing rates between the 'excited' groups is underwhelming, particularly when there are more neurons in the group with the smaller average. It seems some other analysis would fit better here. Maybe a comparison of the empirical cumulative distributions of the firing rates between conditions? As for confusion regarding the analysis performed:

What exactly were the authors comparing here? Is it baseline vs evoked activity for each neuron for each taste?:

'As an initial look at how illness impacts taste-evoked spiking activity of GC neurons, we evaluated the quantity and magnitudes of responses elicited by tastes delivered directly into the mouth via IOC. Paired-sampled t-tests (with Bonferroni correction for multiple comparisons) revealed that LiCl did not significantly impact the magnitude of responses in a taste-specific manner (ps <0.05).'

Wording here is confusing. By putting the (within condition) it sounds like the averages are for each condition. Is it that you took the average differences of activity, compared to baseline, for all tastes for each neuron and performed a Chi square test?:

"For this reason, we averaged taste responses (within condition) and a subsequent chi-square test of independence revealed a significant relationship between condition and direction of response on neuron count"

3. I'm not sure I understood the following argument. Could the authors provide a bit more clarification?:

'While one could speculate that illness' effect on GC μ power merely represents changes in firing rates (FRs) across time, a two-way ANOVA revealed a there was no significant interaction between the experimental group (saline-LiCl versus saline- saline) and quartile on change in FR (F(3, 33) = 2.39, p = 0.09); this suggests that our observed effect on μ is not simply a gating of firing rate direction, but rather a matter of synchrony being modulated.'

4. This may relate to the above. The authors make a clear point that the changes in mu power are transient, yet the observed effects in the paper raise the question about whether a good portion of the observations may be due to changes of evoked taste activity that occurs within mu. The reason I'm suggesting this is that prior work from the Katz group has shown that tastes delivered during mu results in a similar effect which is observed in this paper - that discrimination across similarly palatable tastes decreases while palatability coding increases. This may not necessarily matter to the main point of the paper, but if more tastes are delivered during the mu state, this result would certainly fit with prior work from the lab.

5. In Figure 1A, did the authors forget to include the control group, shouldn't there also be a group of rats with saline injections at the same time as the LiCl injections? If I'm not misunderstanding the design, I would certainly add this since readers might assume that all comparisons were to the NaCl injections on the second day.

6. Figure 2B references in the results section are flipped (top - control; bottom - experimental)

7. In the results, when discussing figure 2C, the authors mention 'subsequent testing'. What exactly was performed here?

8. The authors ran an LDA classifier and compared the results across conditions using a Mann-Whitney U test (page 9). My guess is that a classifier was run for each neuron rather than on a pseudo-ensemble, but it's not clear which is the case either looking in the results or the methods sections. If the latter, please add more clarification about how separate samples, and how many, were included in the Mann-Whitney U-Test. 

9. Editing error: 'a two-way ANOVA revealed a there was no'

# Author comment: Author response to Decision Letter 1

**DOI:** 10.1371/journal.pbio.3001537.r003

2 May 2022

Attachment Submitted filename: PLOS Authors response_Final.docx Click here for additional data file.

# Peer review: Decision Letter 2

**DOI:** 10.1371/journal.pbio.3001537.r004

**Authors:** Kris Dickson

24 May 2022

Dear Dr Katz,

Thank you for your patience while we considered your revised manuscript "LiCl-induced sickness modulates field potential spectral power and response dynamics in rat gustatory cortex." for consideration as a Research Article at PLOS Biology. Your revised study has now been evaluated by the PLOS Biology editors, the Academic Editor and the original reviewers. 

In light of the reviews, which you will find at the end of this email, we are happy to continue consideration of your study at PLOS Biology but do not feel that additional revisions are still needed based on feedback we've received from Reviewers 1 and 2. In particular, Reviewer 1 continues to have concerns with the experimental set-up looking at distinct cohorts of rats, and has asked if you can provide more direct evidence of a change in behavior related to illness in order to rule out that LiCl itself is responsible for the changes in neural activity. We ask that you address this concern as best you are able, and address the additional issues raised by Reviewer 1 and Reviewer 2. We will then assess your revised manuscript and your response to the reviewers' comments with our Academic Editor. We will aim to avoid further rounds of peer-review, although we might need to consult with Reviewer 1 depending on the nature of the revisions.

We expect to receive your revised manuscript within 1 month. Please email us (plosbiology@plos.org) if you have any questions or concerns, or if you feel you would need an extension in order to fully address the remaining concerns. 

At this stage, your manuscript remains formally under active consideration at our journal; please notify us by email if you do not intend to submit a revision so that we withdraw the manuscript.

**IMPORTANT - SUBMITTING YOUR REVISION**

Your revisions should address the specific points made by each reviewer. Please submit the following files along with your revised manuscript:

1. A 'Response to Reviewers' file - this should detail your responses to the editorial requests, present a point-by-point response to all of the reviewers' comments, and indicate the changes made to the manuscript. 

*NOTE: In your point-by-point response to the reviewers, please provide the full context of each review. Do not selectively quote paragraphs or sentences to reply to. The entire set of reviewer comments should be present in full and each specific point should be responded to individually.

You should also cite any additional relevant literature that has been published since the original submission and mention any additional citations in your response. 

2. In addition to a clean copy of the manuscript, please also upload a 'track-changes' version of your manuscript that specifies the edits made. This should be uploaded as a "Revised Article with Changes Highlighted " file type. 

*Resubmission Checklist*

When you are ready to resubmit your revised manuscript, please refer to this resubmission checklist: https://plos.io/Biology_Checklist

To submit a revised version of your manuscript, please go to https://www.editorialmanager.com/pbiology/ and log in as an Author. Click the link labelled 'Submissions Needing Revision' where you will find your submission record. 

Please make sure to read the following important policies and guidelines while preparing your revision:

*Published Peer Review*

Please note while forming your response, if your article is accepted, you may have the opportunity to make the peer review history publicly available. The record will include editor decision letters (with reviews) and your responses to reviewer comments. If eligible, we will contact you to opt in or out. Please see here for more details:

https://blogs.plos.org/plos/2019/05/plos-journals-now-open-for-published-peer-review/

*PLOS Data Policy*

Please note that as a condition of publication PLOS' data policy (http://journals.plos.org/plosbiology/s/data-availability) requires that you make available all data used to draw the conclusions arrived at in your manuscript. If you have not already done so, you must include any data used in your manuscript either in appropriate repositories, within the body of the manuscript, or as supporting information (N.B. this includes any numerical values that were used to generate graphs, histograms etc.). For an example see here: http://www.plosbiology.org/article/info%3Adoi%2F10.1371%2Fjournal.pbio.1001908#s5

*Blot and Gel Data Policy*

We require the original, uncropped and minimally adjusted images supporting all blot and gel results reported in an article's figures or Supporting Information files. We will require these files before a manuscript can be accepted so please prepare them now, if you have not already uploaded them. Please carefully read our guidelines for how to prepare and upload this data: https://journals.plos.org/plosbiology/s/figures#loc-blot-and-gel-reporting-requirements

*Protocols deposition*

To enhance the reproducibility of your results, we recommend that if applicable you deposit your laboratory protocols in protocols.io, where a protocol can be assigned its own identifier (DOI) such that it can be cited independently in the future. Additionally, PLOS ONE offers an option for publishing peer-reviewed Lab Protocol articles, which describe protocols hosted on protocols.io. Read more information on sharing protocols at https://plos.org/protocols?utm_medium=editorial-email&utm_source=authorletters&utm_campaign=protocols

Thank you again for your submission to our journal. We hope that our editorial process has been constructive thus far, and we welcome your feedback at any time. Please don't hesitate to contact us if you have any questions or comments.

Sincerely,

Kris

Kris Dickson, Ph.D. (she/her)

Neurosciences Senior Editor/Section Manager

PLOS Biology

kdickson@plos.org

----------------------------------------------------------------

REVIEWS:

Reviewer's Responses to Questions

PLOS authors have the option to publish the peer review history of their article (what does this mean?). If published, this will include your full peer review and any attached files.

If you choose “no”, your identity will remain anonymous but your review may still be made public.

**Do you want your identity to be public for this peer review?** For information about this choice, including consent withdrawal, please see our Privacy Policy.

Reviewer #1: No

Reviewer #2: No

Reviewer #3: No

Reviewer #4: Yes: Matthew Gardner

Reviewer #1: I thank the Authors for addressing the majority of my concerns. The improved manuscript was significantly easier to understand. However, I do not believe a key concern was adequately addressed. That is, the supposition that the injection of LiCl induced illness/sickness/gastrointestinal malaise in the rat's subjected to neural recordings. 

I do not believe this assumption to be insignificant, since a majority of the results and discussion focus on the results from this group. I do not agree with the Authors position, as indicated by their response to a similar critique raised by Reviewer 3, that changes in neural activity and behavior in separate groups of rats is "more conservative and compelling when reached on the basis of datasets that were collected separately." What would be compelling is showing the relationship between changes in neural activity and behavior within the same animals. In the methods, the Authors state that "Three cameras (positioned below, above, and diagonally within the behavioral chamber (see Apparatus)" were used to record mobility in a separate behavior group. Were these cameras used to record behavior during neural recording sessions? Can the Authors do a similar analysis of rearing behavior (Figure 3) for the neural recording group? 

Without evidence of a change in behavior related to illness, one could simply argue that LiCl itself is responsible for the changes in neural activity. If the Authors cannot provide evidence supporting their assumption that the rats in the neural recording group were in fact made sick by the LiCl-injection, they should temper their use of terms like "illness" or "sickness" when describing the effects induced by LiCl-injection. However, I will acquiesce if the Editor and other Reviewers feel that the behavioral data provided showing that a LiCl-injection modified rearing behavior in a separate group of rats is sufficient to support the declaration that the rats in the neural recording group were made sick by a similar injection of LiCl.

Additional concerns:

The newly provided representative PSTHs in Figure 4 raise some questions. The authors posit that taste information is not represented within the first 200ms. However, three of the four representative examples clearly show that tastes evoke different responses during the first 200ms. Furthermore, the authors state that there was no overall difference in baseline activity between Saline and LiCl sessions. However, the baseline activity for the inhibitory response shows a baseline response of ~6Hz on Saline day and ~18Hz on LiCl day, a three-fold increase. Is the y-axis on the LiCl day correct? It would be helpful to the reader if the PSTHs used the same scale. 

It's possible that these examples are outliers and do not represent the population responses described by the Authors. If so, I suggest they choose different representative examples that align with the broader data set. If these examples are in fact representative, then the authors must reanalyze their data to account for the taste-evoked activity occurring within the first 200ms.

Reviewer #2: Overall, the authors have addressed my previous concern in an adequate manner. 

It would be helpful to know some more information about the new figure 4. I must admit that this figure confused me for two reasons. In the legend, the authors stated that in panel A "...(PSTH) for pairs of representative neurons....". 1) If they are pairs, why are the y axes (for the two PSTHs in the top row and for the two PSTHs in the bottom row) not the same? 2) If these are representative neurons, why is the neural activity evoked by the different stimuli in the first 200 ms clearly different for 3/4 of the PSTHs? Based on the authors' claims (in this and other manuscripts), there should be no chemosensory activity in this early time window. If the stimulus-specific differences observed in the PSTHs of the new Fig. 4 are not chemosensory in nature, what can explain them? In the method section, the authors say that the activity in the first 200ms reflect tactile stimulation of the tongue. Thus, I was expecting to see the responses evoked by the different stimuli within the first 200ms to be mostly overlapping. I admit that this is not a major issue (unlike the concerns raised by two other reviewers about the lack of behavioral signatures of mild sickness, or malaise, in the electrophysiological group). However, in light of the new Fig. 4, the rationale of ignoring the first 200ms of post-taste activity for some analysis is weaker and can create confusion for the reader. One option could be to change the representative neurons showed in Fig. 4 or, even better, to include the first 200ms in all analysis.

Reviewer #3: The authors answered my questions. 

Reviewer #4: The authors fully addressed all of my concerns.

# Author comment: Author response to Decision Letter 2

**DOI:** 10.1371/journal.pbio.3001537.r005

8 Jun 2022

Attachment Submitted filename: Stone et al - Response letter 2.docx Click here for additional data file.

# Editor report: Decision Letter 3

**DOI:** 10.1371/journal.pbio.3001537.r006

**Authors:** Kris Dickson

17 Jun 2022

Dear Dr Katz,

Thank you for your patience while we considered your revised manuscript "Systemic administration of illness-inducing LiCl modulates field potential spectral power and response dynamics in rat gustatory cortex." for publication as a Research Article at PLOS Biology. This revised version of your manuscript has been evaluated by the PLOS Biology editors and the Academic Editor.

I am happy to inform you that, based on our editorial evaluation of your revisions, we are likely to accept this manuscript for publication. At this stage, we simply need you to satisfactorily address the necessary data and other policy-related requests.

In addition, we'd ask that you consider a possible title change to ensure the study is more broadly accessible to our wide audience. Maybe something along the lines of the following (appreciating that this might be too simplistic and not convey all of the nuances you are aiming for...):

Sickness modulates rat cortical taste responses

LiCl-induced sickness modulates rat gustatory cortical responses

As you address the data and policy items, please take this last chance to review your reference list to ensure that it is complete and correct. If you have cited papers that have been retracted, please include the rationale for doing so in the manuscript text, or remove these references and replace them with relevant current references. Any changes to the reference list should be mentioned in the cover letter that accompanies your revised manuscript.

We expect to receive your revised manuscript within two weeks. 

To submit your revision, please go to https://www.editorialmanager.com/pbiology/ and log in as an Author. Click the link labelled 'Submissions Needing Revision' to find your submission record. Your revised submission must include the following:

- a cover letter that should detail your responses to any editorial requests, if applicable, and whether changes have been made to the reference list

- a Response to Reviewers file that provides a detailed response to the reviewers' comments (if applicable)

- a track-changes file indicating any changes that you have made to the manuscript. 

NOTE: If Supporting Information files are included with your article, note that these are not copyedited and will be published as they are submitted. Please ensure that these files are legible and of high quality (at least 300 dpi) in an easily accessible file format. For this reason, please be aware that any references listed in an SI file will not be indexed. For more information, see our Supporting Information guidelines:

https://journals.plos.org/plosbiology/s/supporting-information

*Published Peer Review History*

Please note that you may have the opportunity to make the peer review history publicly available. The record will include editor decision letters (with reviews) and your responses to reviewer comments. If eligible, we will contact you to opt in or out. Please see here for more details:

https://blogs.plos.org/plos/2019/05/plos-journals-now-open-for-published-peer-review/

*Press*

Should you, your institution's press office or the journal office choose to press release your paper, please ensure you have opted out of Early Article Posting on the submission form. We ask that you notify us as soon as possible if you or your institution is planning to press release the article.

*Protocols deposition*

To enhance the reproducibility of your results, we recommend that if applicable you deposit your laboratory protocols in protocols.io, where a protocol can be assigned its own identifier (DOI) such that it can be cited independently in the future. Additionally, PLOS ONE offers an option for publishing peer-reviewed Lab Protocol articles, which describe protocols hosted on protocols.io. Read more information on sharing protocols at https://plos.org/protocols?utm_medium=editorial-email&utm_source=authorletters&utm_campaign=protocols

Please do not hesitate to contact me should you have any questions.

Sincerely,

Kris

Kris Dickson, Ph.D. (she/her)

Neurosciences Senior Editor/Section Manager,

kdickson@plos.org,

PLOS Biology

------------------------------------------------------------------------

DATA POLICY:

You may be aware of the PLOS Data Policy, which requires that all data be made available without restriction: http://journals.plos.org/plosbiology/s/data-availability. For more information, please also see this editorial: http://dx.doi.org/10.1371/journal.pbio.1001797

Note that we do not require all raw data. Rather, we ask that all individual quantitative observations that underlie the data summarized in the figures and results of your paper be made available. We appreciate that you have indicated that data are fully available from the first or last authors. However, we require that the data also be provided in one of the following forms:

1) Supplementary files (e.g., excel). Please ensure that all data files are uploaded as 'Supporting Information' and are invariably referred to (in the manuscript, figure legends, and the Description field when uploading your files) using the following format verbatim: S1 Data, S2 Data, etc. Multiple panels of a single or even several figures can be included as multiple sheets in one excel file that is saved using exactly the following convention: S1_Data.xlsx (using an underscore).

2) Deposition in a publicly available repository (e.g. Zenodo etc). Please also provide the accession code or a reviewer link so that we may view your data before publication. 

Regardless of the method selected, please ensure that you provide the individual numerical values that underlie the summary data displayed in the following figure panels as they are essential for readers to assess your analysis and to reproduce it:

Fig 2B,C; Fig 3B; Fig 4A-C; Fig 5; Fig 6A,B; Fig 7B; Fig 8a-E

NOTE: the numerical data provided should include all replicates AND the way in which the plotted mean and errors were derived (it should not present only the mean/average values).

***Please also ensure that FIGURE LEGENDS in your manuscript include information on where the underlying data can be found, and ensure your supplemental data file/s has a legend. (NB: This step is often forgotten!!!)

***Please ensure that your Data Statement in the submission system accurately describes where your data can be found.

------------------------------------------------------------------------

SPECIES INDICATED IN THE ABSTRACT? 

- Please note that per journal policy, the model system/species studied should be clearly stated in the abstract of your manuscript. 

------------------------------------------------------------------------

DATA NOT SHOWN?

- Please note that per journal policy, we do not allow the mention of "data not shown", "personal communication", "manuscript in preparation" or other references to data that is not publicly available or contained within this manuscript. Please either remove mention of these data or provide figures presenting the results and the data underlying the figure(s).

------------------------------------------------------------------------

Reviewer remarks: N/A

# Editor report: Decision Letter 4

**DOI:** 10.1371/journal.pbio.3001537.r007

**Authors:** Kris Dickson

29 Jun 2022

Dear Dr Katz,

Thank you for the submission of your revised Research Article "LiCl-induced sickness modulates rat gustatory cortical responses" for publication in PLOS Biology. On behalf of my colleagues and the Academic Editor, Thorsten Kahnt, I am pleased to say that we can in principle accept your manuscript for publication, provided you address any remaining formatting and reporting issues. These will be detailed in an email you should receive within 2-3 business days from our colleagues in the journal operations team; no action is required from you until then. Please note that we will not be able to formally accept your manuscript and schedule it for publication until you have completed any requested changes.

Please take a minute to log into Editorial Manager at http://www.editorialmanager.com/pbiology/, click the "Update My Information" link at the top of the page, and update your user information to ensure an efficient production process.

PRESS

We frequently collaborate with press offices. If your institution or institutions have a press office, please notify them about your upcoming paper at this point, to enable them to help maximize its impact. If the press office is planning to promote your findings, we would be grateful if they could coordinate with biologypress@plos.org. If you have previously opted in to the early version process, we ask that you notify us immediately of any press plans so that we may opt out on your behalf.

We also ask that you take this opportunity to read our Embargo Policy regarding the discussion, promotion and media coverage of work that is yet to be published by PLOS. As your manuscript is not yet published, it is bound by the conditions of our Embargo Policy. Please be aware that this policy is in place both to ensure that any press coverage of your article is fully substantiated and to provide a direct link between such coverage and the published work. For full details of our Embargo Policy, please visit http://www.plos.org/about/media-inquiries/embargo-policy/.

Thank you again for choosing PLOS Biology for publication and supporting Open Access publishing. We look forward to publishing your study. 

Sincerely, 

Kris

Kris Dickson, Ph.D. (she/her)

Neurosciences Senior Editor/Section Manager

PLOS Biology

kdickson@plos.org
